# Regulation of the cholesterol biosynthetic pathway and its integration with fatty acid biosynthesis in the oleaginous microalga *Nannochloropsis oceanica*

**DOI:** 10.1186/1754-6834-7-81

**Published:** 2014-05-30

**Authors:** Yandu Lu, Wenxu Zhou, Li Wei, Jing Li, Jing Jia, Fei Li, Steven M Smith, Jian Xu

**Affiliations:** 1 Single-Cell Center, CAS Key Laboratory of Biofuels and Shandong Key Laboratory of Energy Genetics, Qingdao Institute of BioEnergy and Bioprocess Technology, Chinese Academy of Sciences, Qingdao, Shandong 266101, China; 2 Australian Research Council, Centre of Excellence in Plant Energy Biology, The University of Western Australia, 35 Stirling Highway, Crawley, WA 6009, Australia

**Keywords:** Cholesterol biosynthetic pathway, Fatty acid biosynthesis, Feedback regulation, *Nannochloropsis*

## Abstract

**Background:**

Sterols are vital structural and regulatory components in eukaryotic cells; however, their biosynthetic pathways and functional roles in microalgae remain poorly understood.

**Results:**

In the oleaginous microalga *Nannochloropsis oceanica*, the sterol biosynthetic pathway produces phytosterols as minor products and cholesterol as the major product. The evidence together with their deduced biosynthetic pathways suggests that *N. oceanica* exhibits features of both higher plants and mammals. Temporal tracking of sterol profiles and sterol-biosynthetic transcripts in response to changes in light intensity and nitrogen supply reveal that sterols play roles in cell proliferation, chloroplast differentiation, and photosynthesis. Furthermore, the dynamics of fatty acid (FA) and FA-biosynthetic transcripts upon chemical inhibitor-induced sterol depletion reveal possible co-regulation of sterol production and FA synthesis, in that the squalene epoxidase inhibitor terbinafine reduces sterol content yet significantly elevates free FA production. Thus, a feedback regulation of sterol and FA homeostasis is proposed, with the 1-deoxy-D-xylulose 5-phosphate synthase (DXS, the committed enzyme in isoprenoid and sterol biosynthesis) gene potentially subject to feedback regulation by sterols.

**Conclusion:**

These findings reveal features of sterol function and biosynthesis in microalgae and suggest new genetic engineering or chemical biology approaches for enhanced oil production in microalgae.

## Background

Sterols are vital components of all eukaryotic cells [1]. In higher plants, they play a structural role in cell viability, embryogenesis, pattern formation, cell division, chloroplast biogenesis, and modulation of activity and distribution of membrane-bound proteins such as enzymes and receptors [2,3]. In addition, sterols are precursors for many signaling molecules that regulate growth and development in plants and animals, such as insect ecdysteroid molting hormones [4], mammalian steroid hormones [5], and plant brassinosteroid (BR) hormones [6].

Sterols belong to a class of isoprenoids derived from isopentenyl pyrophosphate (IPP), a universal precursor of isoprenoids. In animals and fungi, the cytoplasmic mevalonic acid (MVA) pathway is the only route for biosynthesis of IPP, the building block for lanosterol, which is then metabolized into cholesterol in animals and ergosterol in fungi [7]. In higher plants, IPP can be derived via either the MVA pathway or the plastidial 1-deoxyxylulose 5-phosphate or methylerythritol phosphate (MEP) pathway, despite the former being the main contributor to sterol biosynthesis [8,9]. In *Arabidopsis*, sterol biosynthetic mutants can be classified into two distinct groups: BR-independent mutants, which are defective in genes in the pathway from cycloartenol to 24-methylenelophenol [10], and BR-dependent mutants, which are defective in genes at the latter part of the sterol biosynthetic pathway (from 24-methylenelophenol to campesterol, which is considered as the precursor for BRs) [11]. Besides serving as a BR precursor, sterols appear to play distinct signaling functions during plant development, since phenotypes of sterol biosynthetic mutants cannot be rescued by addition of exogenous BR [12].

Co-regulation of sterol synthesis and fatty acid (FA) production is essential for maintaining the biosynthesis-versus-turnover balance of membranes during cellular growth [13]. In animals, sterols and FAs are coordinately regulated by a feedback system mediated by a conserved family of transcription factors called sterol regulatory element binding proteins (SREBPs), which controls a cascade of biosynthetic enzymes for endogenous cholesterol, FA, triacylglycerol (TAG), and phospholipid [14,15]. Activated SREBP binds to sterol response elements in the promoter and/or enhancer regions of target genes and induces transcription of at least 30 cholesterol- and lipid-synthesis genes (particularly those encoding rate-limiting enzymes, such as hydroxy-methyl-glutaryl-CoA reductase, HMGR and type I FA synthase, FAS) [16]. Manipulation of this regulatory cascade in transgenic mice resulted in 6- and 22-fold increases in cholesterol content and TAG content, respectively, and consequentially massive fatty livers [17].

Microalgae are promising feedstock for sustainable and scalable production of biofuel [18]; however, few microalgal strains found in nature are endowed with the wide array of traits demanded by a large-scale and economically competitive production scheme [19]. It is therefore urgent to identify molecules and mechanisms that regulate microalgal growth, development, and stress responses for strain improvement. Sterols in microalgae display enormous diversity due to the high degree of phylogenetic heterogeneity, the vast number of genera, and the long evolutionary distance among many of them [20,21]. It has long been shown that in microalgae sterol composition varies upon changes in growth stage, light spectrum, or temperature, suggesting important yet largely unknown roles of sterols [22]. Therefore, manipulation of sterol biosynthesis and regulation offers potential for engineering lipid production. Exploration of the sterol-dependent lipogenesis regulatory mechanism in microalgae might provide novel strategies and targets for enhanced lipid production in microalgae. However, little is known about the roles, biosynthesis, and regulation of sterols in microalgae and in particular, whether and how sterol and FA metabolism is co-regulated.

*Nannochloropsis* spp. are a genus of unicellular photosynthetic microalgae belonging to the heterokonts. They are distributed widely in the marine environment as well as in fresh and brackish waters. These algae are of industrial interest because they grow rapidly and can synthesize large amounts of TAG and high-value polyunsaturated FA (for example, eicosapentaenoic acid) [23]. The genomes of multiple species of oleaginous *Nannochloropsis* spp. have been sequenced and annotated [23-27]. Employing an oleaginous industrial microalga *N. oceanica* IMET1 as a model, this study has aimed to determine the sterol composition and biosynthetic pathway in microalgae, to investigate the role of sterol biosynthesis in photosynthesis and growth, to study the influence of light and nitrogen supply, and to probe the effects of sterol levels on FA accumulation. Our findings expand the understanding of sterol function in microalgae and should assist rational genetic or process engineering for microalgae-based production of biofuels or other value-added bioproducts.

## Results

### *N. oceanica* sterol biosynthetic pathway shares features in structure and sterol profiles with those of animals and plants

**Figure 1:**
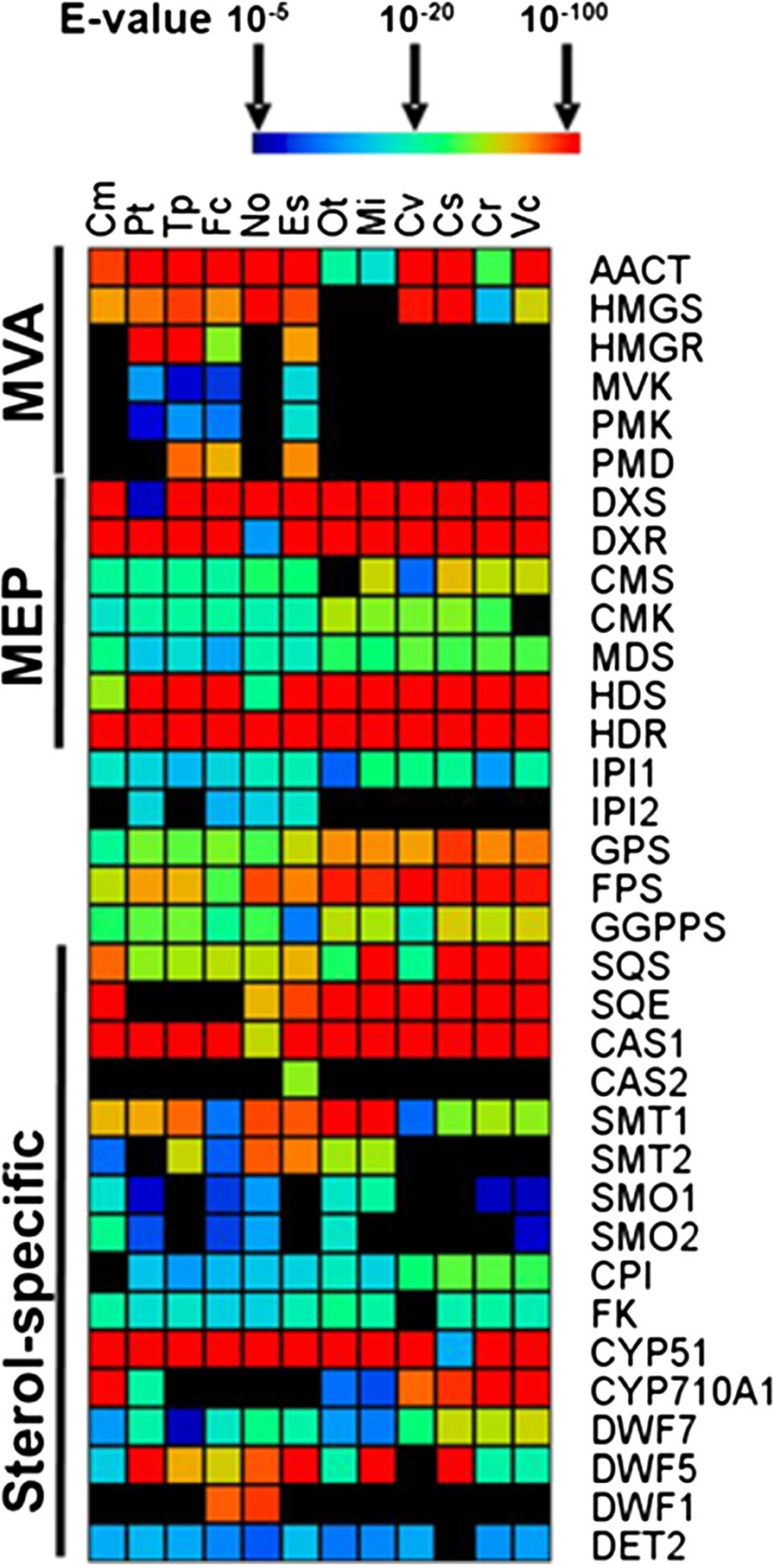
**Conservation of sterol biosynthetic genes in eukaryotic algae.** The color key (top) indicates the similarity of the gene to the closest match and ranges from low similarity (black) to high similarity (red). Black areas indicate no Blastp hit below the applied e-value threshold (1e-5). Red areas indicate orthologs with Blastp e-values below 1e-100. Color in the heatmap is scaled column-wise based on the bit values of tblastn results (Additional file 1). Abbreviations: red alga *Cyanidioschyzon merolae* (Cm), diatom *Phaeodactylum tricornutum* (Pt), diatom *Thalassiosira pseudonana* (Tp), diatom *Fragilariopsis cylindrus* (Fc), eustigmatophyte *N. oceanica* (No), brown alga *Ectocarpus siliculosus* (Es), green alga *Ostreococcus tauri* (Ot), green alga *Micromonas* sp. RCC299 (Mi), green alga *Chlorella variabilis* NC64A (Cv), green alga *Coccomyxa subellipsoidea* C-169 (Cs), green alga *Chlamydomonas reinhardtii* (Cr), and green alga *Volvox carteri* (Vc). See Additional file 2: Figure S1 for the phylogenetic tree of the sampled species.

Among different organisms, the core sterol biosynthetic pathway consists of a common set of enzymes that exhibit strong conservation in amino acid sequences; however the pathway architecture and substrate specificity can vary significantly [1]. *In silico* reconstruction and comparison of sterol biosynthetic pathways among 12 selected algal species revealed intriguing structural features of the *N. oceanica* pathway, which include characteristics from both higher plants and animals (Figure 1 and Additional file 1).

The sterol synthetic pathway of *N. oceanica* includes higher plant-like features. Higher plants have two sterol methyltransferase (SMT) enzymes that use different substrates to give either methylated (SMT1) or ethylated (SMT2) phytosterols. In the *N. oceanica* genome, two candidate genes encoding SMT were identified, which resemble those of higher plants in primary sequence. In contrast, the diatom *Phaeodactylum tricornutum* and several green algae including *Chlamydomonas reinhardtii*, *Chlorella variabilis* NC64A, *Coccomyxa subellipsoidea* C-169, and *Volvox carteri* have a single candidate gene encoding SMT (Figure 1, see Additional file 2: Figure S1 for the phylogenetic tree of the sampled species) that potentially catalyzes successive methylation reactions to give methylated and ethylated products.

Features that are shared with animals were also present in the sterol synthetic pathway of *N. oceanica*. In the sampled microalgae, the key enzyme catalyzing sterol side chain reduction is different from that of *Arabidopsis* and higher plants in general. In higher plants, the enzyme, namely sterol 24(28) isomerase-reductase, is encoded by the *DWF1* gene in *Arabidopsis* and performs dual functions. It catalyzes C-24(28) double bond isomerization to form a 24(25) double bond, followed by reduction of the 24(25) double bond. In animals and yeast, the equivalent enzymes are 24-dehydrocholesterol reductase (DHCR24) and sterol C-24(28) reductase (ERG4), which only catalyze the reduction reaction. DWF1 or DHCR24 orthologs have not been found in algae except in *N. oceanica* and the diatom *Fragilariopsis cylindrus*. Amino acid sequence analysis indicates that *N. oceanica* sterol 24(25) reductase is clustered with that of choanoflagellates (the closest living unicellular relatives of animals [28]) and has greater similarity to animal DHCR24 than to higher plant DWF1 (Additional file 2: Figure S2). The evidence based on DWF1/DHCR24 therefore suggests features of an animal-type sterol biosynthetic pathway.

To test these predicted features of the sterol biosynthetic pathway, we characterized the chemical profile of sterols in *N. oceanica* IMET1, which unveiled an animal-like composition of sterols. In *N. oceanica*, five sterols were identified (sterol structures indicated as bold numbers in Additional file 2: Figure S3 and in the following text). Cholesterol (**2**) is the most abundant sterol, comprising 70% to 75% of the total (Table 1). The remaining sterols are fucosterol (**11**), isofucosterol (**13**), 24-methylcholesta-5, 25(27)-dienol (**10**), and 24-methylenecholesterol (**7**) (Additional file 2: Figure S3). In *N. oceanica*, sterols with a C-22 double bond are not found, supporting the absence of CYP710A (a C-22 desaturase; Figure 1). Although a protein (g4528) with similarity to CYP710A was present, its primary sequence is closer to that of CYP51 than to CYP710A (Additional file 1). The lack of CYP710A is a typical feature of animals, as higher plant genomes usually encode highly conserved CYP710A. In addition, the side-chain double bond formed by SMTs is retained, supporting the presence of an animal-type DHCR24 (Additional file 2: Figure S2). This is further supported by the accumulation of a large amount of cholesterol in *N. oceanica* (Table 1), which is the sole sterol in animals. On the other hand, only a minor amount of the phytosterols, which are the dominant forms of sterols in higher plants, was found in *N. oceanica* (Table 1). Therefore, *N. oceanica* sterol profiles exhibit features of both animals and higher plants.

**Table 1:** **GC/MS data for sterol profiling from *N. oceanica *** **treated with sterol biosynthetic inhibitors**

**Sterols**	**Structure**	**RT (min)**	**RRTc**	**Sterol profile (%)**					
				**Control**	**CLO**	**TBF**	**TEB**	**TDM**	**25-AZA**
Squalene	**1**	10.25	0.755	-*	-	12.0	-	-	-
Cholesterol	**2**	13.58	1.000	74.0	70.4	61.6	62.0	56.2	81.3
Cholest-8-enol	**3**	13.75	1.012	-	-	-	-	8.2	-
Desmosterol	**4**	13.91	1.024	-	-	-	-	-	3.0
Pollinastanol	**5**	14.33	1.056	-	-	-	-	13.1	-
4, 14-Dimethylcholest-8-enol	**6**	14.53	1.070	-	-	-	1.7	-	-
24-Methylenecholesterol	**7**	14.59	1.074	1.4	2.5	0.7	2.7	-	4.0
24(25)-Dihydrolanosterol	**8**	15.18	1.117	-	-	-	1.7	-	-
4-Methylpollinasterol	**9**	15.47	1.139	-	-	-	-	3.7	-
24-Methylcholesta-5, 25(27)-dienol	**10**	15.51	1.142	0.9	1.0	0.9	0.8	0.3	1.0
Fucosterol	**11**	15.68	1.155	10.1	10.1	10.3	7.2	6.8	6.0
Obtusifoliol	**12**	15.73	1.158	-	-	-	0.2	-	-
Isofucosterol	**13**	15.91	1.171	13.5	16.1	14.3	8.9	9.3	4.8
Stigmasta-8, 24(28) *E*-dienol	**14**	15.93	1.173	-	-	-	-	0.4	-
Cycloartenol	**15**	16.06	1.182	-	-	-	12.8	-	-
Stigmasta-8, 24(28) Z-dienol	**16**	16.16	1.190	-	-	-	-	1.9	-
Cycloartanol	**17**	16.59	1.222	-	-	-	2.1	-	-

Cultures in exponential phase were diluted in fresh media and grown for 12 h under 50 μmol photons m^-2^ s^-1^ light. Subsequently, cultures were treated with inhibitors at a concentration of IC_50_ or an equivalent amount of DMSO (not exceeding a final concentration of 0.1%). Cell aliquots were collected for sterol profiling four days later. Three biological replicates of algal cultures were established under each treatment. Abbreviations: RRTc, relative retention time to cholesterol; CLO, clomazone; TBF, terbinafine; TEB, tebuconazole; TDM, tridemorph; 25-AZA, 25-azalanosterol. The asterisk (*) indicates a value below the detection limit under current experimental conditions. The structure numbers in boldface are listed in Additional file 2: Figure S3.

**Figure 2:**
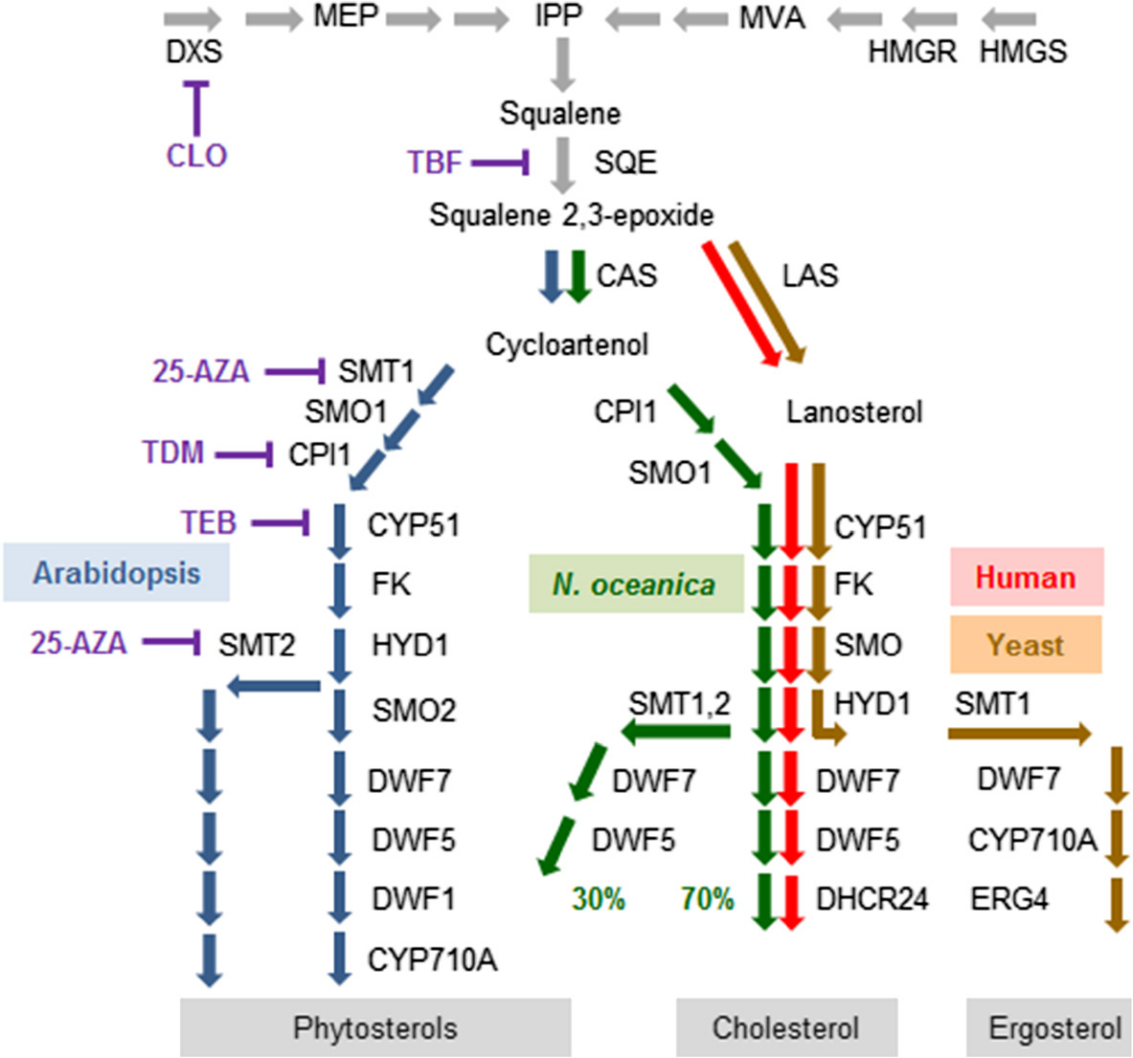
**Deduced pathway of sterol biosynthesis in *N. oceanica*, comparison among plants, humans and yeasts, and sites of action of inhibitors.** Enzyme nomenclature is that commonly used in *Arabidopsis*, because although many human and yeast enzymes have different names and abbreviations, they catalyze equivalent reactions to those of the *Arabidopsis* enzymes. An exception is *Arabidopsis* DWF1 (a dual isomerase-reductase), which is absent in *Nannochloropsis*; instead, this alga has a human-type DHCR24 (the yeast equivalent is ERG4). Full names and functions of enzymes are provided in Additional file 3: Table S1. Lanosterol synthase (LAS) is found in *Arabidopsis* but has a minor role. Enzyme abbreviations: DXS, 1-deoxy-D-xylulose 5-phosphate synthase; HMGR, hydroxy-methyl-glutaryl-CoA reductase; HMGS, hydroxy-methyl-glutaryl-CoA synthase; SQE, squalene epoxidase; CAS, cycloartenol synthase; LAS, lanosterol synthase; SMT, sterol methytransferase; SMO, sterol 4-methyl oxidase; CPI, cycloeucalenol cycloisomerase; CYP51, sterol 14-alpha demethylase; FK, sterol C-14 reductase; HYD, sterol C-8 isomerase; DWF7, delta7 sterol C-5 desaturase; DWF5, sterol C-7 reductase; DWF1, sterol C-24(28) isomerase-reductase; CYP710A, sterol C-22 desaturase; DHCR24, dihydrocholesterol reductase; ERG4, sterol delta 24(28) reductase. Abbreviations for metabolites: MEP, methylerythritol phosphate; IPP, isopentenyl pyrophosphate; MVA, mevalonic acid. Abbreviations for inhibitors: CLO, clomazone; TBF, terbinafine; 25-AZA, 25-azalanosterol; TDM, tridemorph; TEB, tebuconazole.

Furthermore, we adopted a chemical biology approach to probe the architecture of the sterol biosynthetic pathway, in which *N. oceanica* was treated with a series of isoprenoid and sterol biosynthetic inhibitors (SBIs). (See Figure 2 for the target enzymes and Additional file 2: Figure S4 for the inhibition ratios.) The phenotypes of sterol biosynthetic mutants can be mimicked by the application of specific chemical inhibitors, which are powerful tools for elucidating the biosynthesis and functions of sterols [29-33], particularly when a targeted gene knockdown system is not yet available for *N. oceanica* IMET1. The chemical inhibitors we employed here have been well studied and the specificities to their corresponding enzymes established [34-38].

The inhibitor clomazone (CLO) acts on 1-deoxy-D-xylulose 5-phosphate synthase (DXS), a key regulatory enzyme for chloroplast IPP biosynthesis in higher plants [36]. DXS plays an equivalent role to animal HMGR, which is a key regulatory enzyme for cytosolic IPP biosynthesis via the MVA pathway. CLO had a small effect on sterol profiles (Table 1), but it reduced to 70% of the total sterol amount of the control, presumably by limiting IPP supplies to the sterol pathway (Figure 2). Terbinafine (TBF) specifically targets squalene epoxidase (SQE) [34], which is the second committed enzyme of the sterol biosynthetic pathway (Figure 2). It is a critical point for inhibition of sterol biosynthesis, as it reduces sterol content but has no direct effect on the biosynthesis of other isoprenoids [34]. TBF-treated cells accumulated a significant amount of squalene (**1**), supporting the presence and function of SQE (Table 1) and the total amount of sterol was reduced by about 20%. Cells inhibited by tridemorph (TDM), the cycloeucalenol cycloisomerasesterol isomerase (CPI) and sterol C-8 isomerase inhibitor [38], accumulated 9,19-cyclopropyl sterol (pollinastanol, **5**) and delta-8 sterols [obtusifoliol, (**12**), cholest-8-enol (**3**), and stigmasta-8,24(28)-dienols (**14**, **16**)] (Table 1). Thus, CPI is required for sterol biosynthesis, implying that *N. oceanica* uses cycloartenol as a precursor in the biosynthesis of other sterols, consistent with the presence of a cycloartenol synthase (CAS) gene (Figure 1). Cycloartenol has been shown to be the signature sterol in almost all photosynthetic organisms [39]. Treatment with tebuconazole (TEB), an inhibitor of the cytochrome P450 CYP51 [37], resulted in accumulation of 4,4,14-trimethyl (**8**, **15**, **17**) and 4,14-dimethyl sterols (**6**). The relatively large quantities of cycloartanol (**17**) and cycloartenol (**15**) (Table 1) further indicate that cycloartenol is the major precursor for the *N. oceanica* sterol biosynthetic pathway (Figure 2). Moreover, the predominance of sterols without side-chain methylation implies that the 14α-demethylation step occurs before C-24 methylation and C-4 demethylation (Figure 2). This architecture of the biosynthetic pathway differs from that of land plants and is more similar to the cholesterol biosynthetic pathway in animals and the ergosterol biosynthetic pathway in fungi [40]. 25-azalanosterol (25-AZA) is a specific inhibitor of SMT, which determines sterol compositions [35]. Application of 25-AZA resulted in desmosterol (**4**) accumulation (Table 1), revealing a similarity to yeast SMT which exhibits a preference for 4,4-desmethylsterols, unlike other algal or plant SMTs [7].

In summary, the collective findings allowed us to propose a sterol biosynthetic pathway in *N. oceanica* that exhibits both common and distinct features from those in fungi, animals, and green plants (including green algae and land plants) (Figure 2).

### The role of sterols in the growth of *N. oceanica*

**Figure 3:**
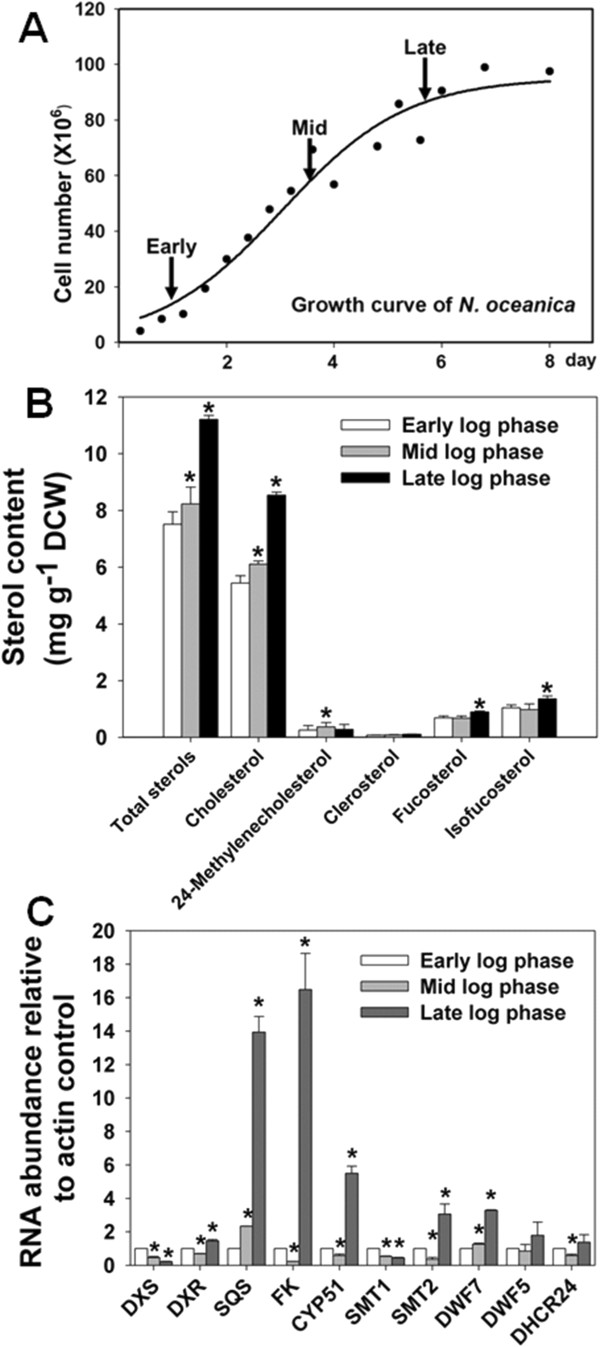
**Chemical analysis and transcripts of sterol biosynthesis during *N. oceanica *growth. (A) **Growth curve of *N. oceanica. ***(B)** Changes in total sterol and sterol composition at different growth stages. **(C)**  Transcript levels of sterol biosynthetic genes at different growth stages. The values are the means of three replicates. Asterisks (*) indicate *P* values < 0.05.

To probe the functional roles of sterols in *N. oceanica*, we next investigated the dynamics of sterol profiles and the expression of their biosynthetic genes during proliferation or cessation of cell division (Figure 3A). Sterol levels exhibited modest increases during the early growth stages of *N. oceanica* cultures, and then increased rapidly at later stages of the culture cycle between 4 × 10^7^ and 10^8^ cells ml^-1^ (Figure 3B). The increase of cholesterol accounts for a significant proportion of the increase in total sterols (Figure 3B). Meanwhile, sterol biosynthetic genes showed a coordinated adaptation in the late culture cycle (Figure 3C). All studied genes except *DXS* and *SMT1* were transcriptionally elevated, with maximum levels at the late log phase. Thus, sterol biosynthesis and accumulation appears to be a feature of late cell growth as the culture approaches stationary phase.

Nitrogen depletion is reported to inhibit cell division in *N. oceanica*[41]. To investigate its effects on sterol biosynthesis, we transferred cells to N-replete and N-depleted media for six days. The sterol level in N-depleted cells was about 33% that of N-replete cells (Additional file 2: Figure S5a). This difference is largely explained by differences in the amount of cholesterol, but surprisingly isofucosterol was disproportionately low, especially compared to fucosterol. This could be explained by differential responses of SMTs to nitrogen depletion. Studies of gene expression during the first 24 h of nitrogen depletion showed that genes of the MEP pathway were appreciably down-regulated (Additional file 2: Figure S5b), which may explain the subsequent decline in sterol levels. While some genes of sterol biosynthesis were initially up-regulated (for example, *FK* and *SMT1*), others (for example, *SMT2* and *DWF5*) were rapidly down-regulated (Additional file 2: Figure S5b). These results suggest a complex response to nitrogen depletion but point to a central role for phytosterols, reflecting the importance of SMTs and potentially the methyl donor S-adenosyl methionine.

### Effects of light on sterol biosynthesis in *N. oceanica*

To probe the relationship between sterol biosynthesis and light, we next examined the responses of sterol profiles and their biosynthetic genes to changes in light intensity. Cultures of *N. oceanica* are typically grown at or below the light saturation point of about 100 μmol photons m^-2^ s^-1^, whereas 300 μmol photons m^-2^ s^-1^ poses a high light stress. High light is known to cause biochemical damage to the photosynthetic system in higher plants, reducing the efficiency of light utilization.

**Figure 4:**
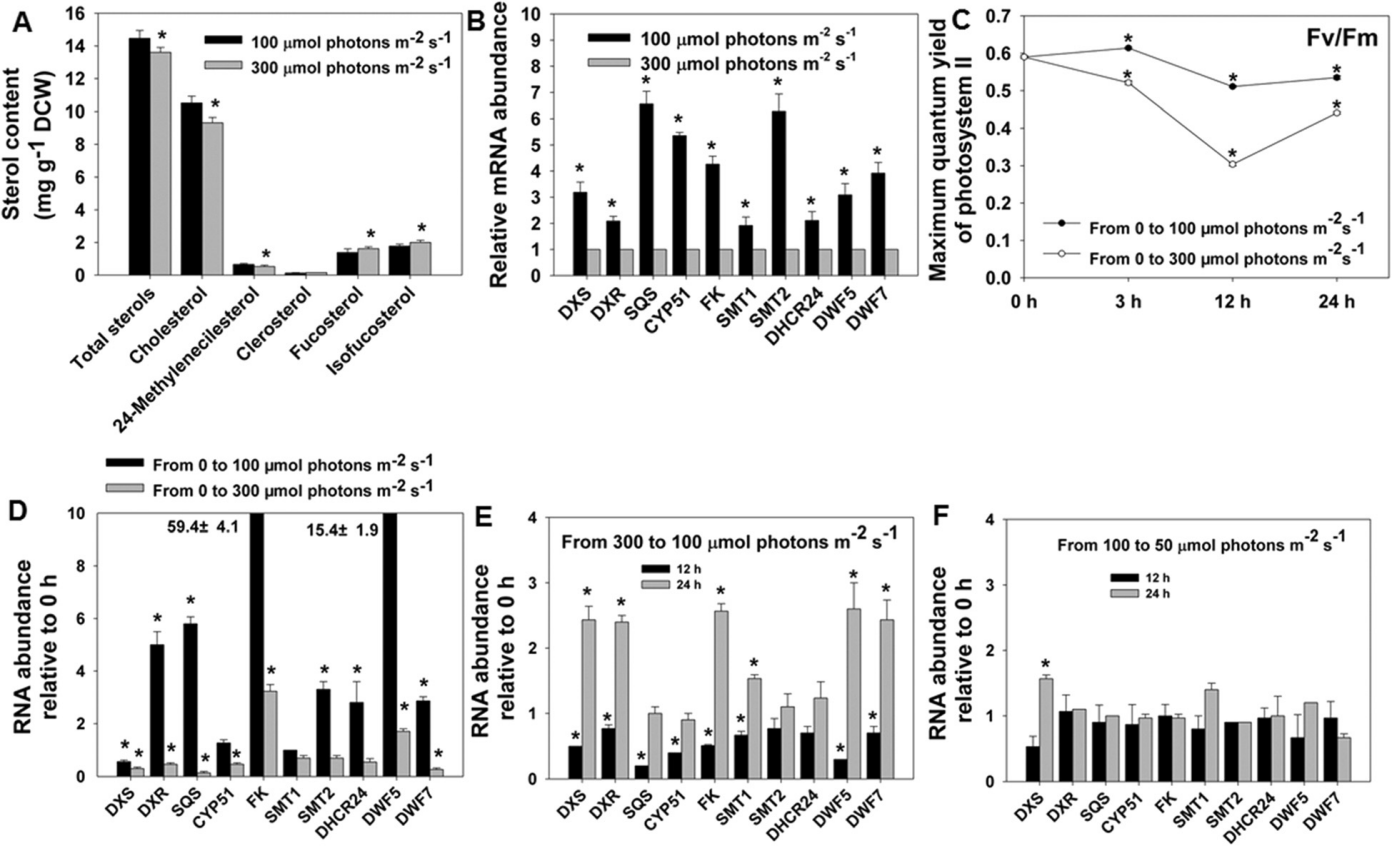
**Transcript levels of sterol metabolic genes of *N. oceanica *in response to changes in light intensity. (A)** Sterol contents under different light intensities. **(B)** Expression of sterol metabolic genes of *N. oceanica* under different light intensities. The transcript levels under 300 μmol photons m^-2^ s^-1^ are normalized to 1.0. Cells at mid-log phase were transferred to constant light intensities of 100 and 300 μmol photons m^-2^ s^-1^. Cells were taken for transcript and sterol analysis after 96 h. **(C)** Maximum photosynthetic efficiency of photosystem II (PSII) of *N. oceanica* in response to the transfer from dark to light. Cells at mid-log phase were transferred to dark for 12 h and then transferred to 300 or 100 μmol photons m^-2^ s^-1^ light. **(D)** Relative mRNA abundance of sterol metabolic genes in *N. oceanica* in response to the transfer from dark to light. **(E)** Relative mRNA abundance of sterol metabolic genes in *N. oceanica* in response to a light intensity change from 300 to 100 μmol photons m^-2^ s^-1^. Cells at mid-log phase were transferred to 300 μmol photons m^-2^ s^-1^ for 12 h, then to 100 μmol photons m^-2^ s^-1^. Samples were collected after 12 and 24 h. **(F)** Relative mRNA abundance of sterol metabolic genes in *N. oceanica* in response to a light intensity change from 100 to 50 μmol photons m^-2^ s^-1^. Cells at mid-log phase were transferred to 100 μmol photons m^-2^ s^-1^ for 12 h, then to 50 μmol photons m^-2^ s^-1.^ Samples were collected after 12 and 24 h. Values are the means of three replicates. Asterisks (*) indicate *P* values < 0.05.

First, cultures with the same cell density were transferred to constant light intensities of 100 and 300 μmol photons m^-2^ s^-1^ for 96 h. RNA and sterols were isolated at the end of the light treatments. The sterol content was lower in cells under 300 μmol photons m^-2^ s^-1^ compared with those under 100 μmol photons m^-2^ s^-1^ (Figure 4A). This is the result of a significantly reduced cholesterol level, whereas phytosterols (fucosterol and isofucosterol) were significantly increased under 300 μmol photons m^-2^ s^-1^ (Figure 4A). This indicates a relationship between light stress and sterol metabolism. All sterol biosynthetic genes examined were expressed at a significantly lower level under 300 μmol photons m^-2^ s^-1^ (Figure 4B). Therefore, in response to high light stress, sterol biosynthesis as a whole is repressed, while some specific phytosterols increase in concentration. This observation is consistent with observations that sterol biosynthesis is modified in the high light response of the green alga *Dunaliella bardawil*[42] and higher plants [43].

To study the short-term effects of high light, cells were first grown under 50 μmol photons m^-2^ s^-1^ to mid-log phase, were then darkness-adapted for 12 h to simulate night, and finally transferred to 100 or 300 μmol photons m^-2^ s^-1^ for 12 h. After this time, the photosynthetic efficiency was measured. Samples were collected for analysis of gene expression at the beginning and the end of the 12-h light treatment. The photosynthetic efficiency was significantly higher at 100 μmol photons m^-2^ s^-1^ than at 300 μmol photons m^-2^ s^-1^ (Figure 4C). The transcript levels of all studied genes were also higher at 100 μmol photons m^-2^ s^-1^ than at 300 μmol photons m^-2^ s^-1^ (Figure 4D). This further indicates that high light treatment rapidly represses sterol biosynthesis genes and impairs photosynthesis.

To determine if such a repression of gene expression is reversible, cells were treated for 12 h at 300 μmol photons m^-2^ s^-1^ and then transferred to 100 μmol photons m^-2^ s^-1^ for 24 h. RNA was isolated at the end of the high light treatment and after 12 and 24 h adaptation to 100 μmol photons m^-2^ s^-1^, for analysis of gene expression. The results showed that expression of sterol biosynthesis genes increased significantly after 24 h at 100 μmol photons m^-2^ s^-1^ (Figure 4E). Thus, sterol biosynthesis gene expression is highly responsive to such light treatments, implying a key role for sterols in adaptation to high light. For comparison, transfer of cells from 100 to 50 μmol photons m^-2^ s^-1^ resulted in only minor changes in expression of sterol biosynthesis genes after 12 and 24 h (Figure 4F), implying that sterol biosynthesis does not play a major role in adaptation to light levels below the saturation point.

### Inhibition of sterol biosynthesis in *N. oceanica* leads to depressed photosynthetic efficiency

**Figure 5:**
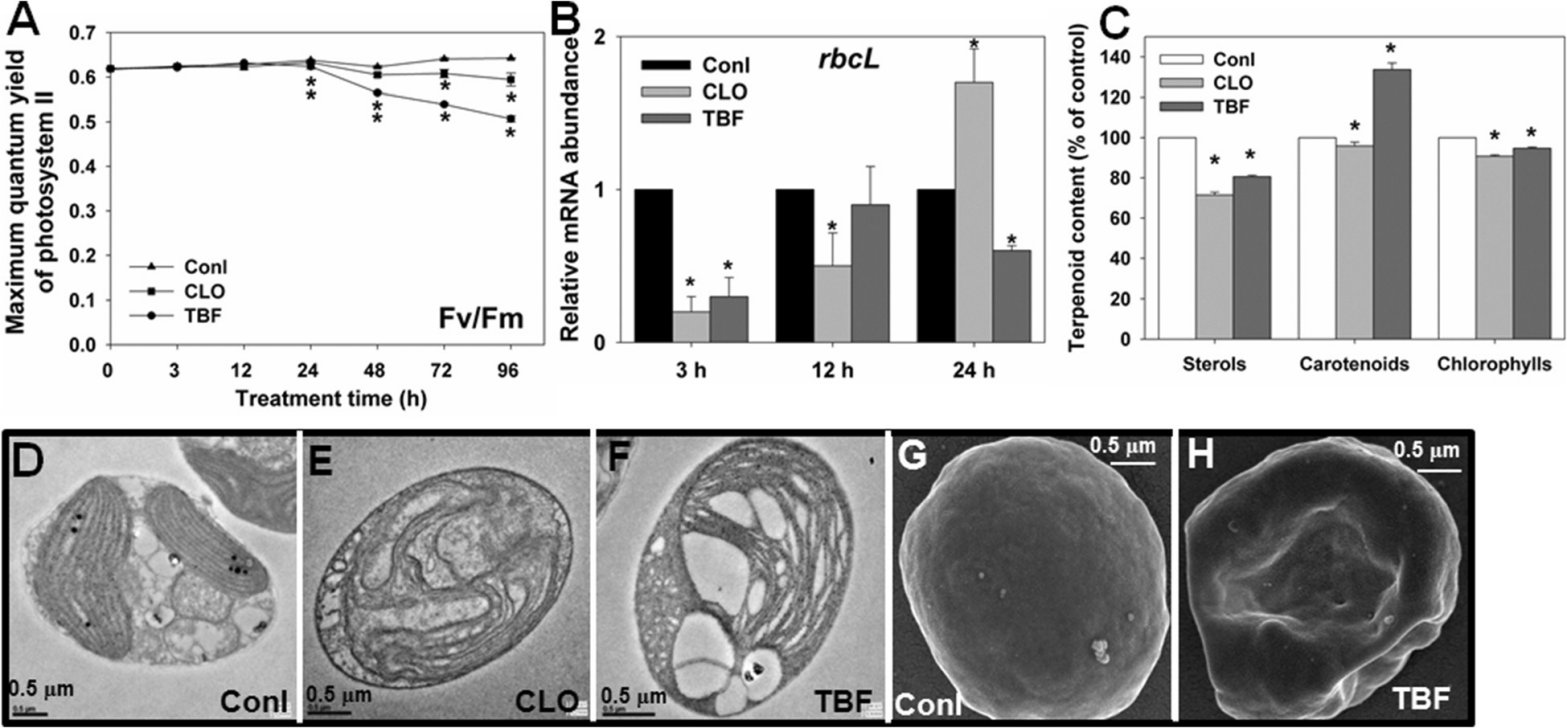
**The consequence of inhibition of sterol biosynthesis on the photosynthetic activity and apparatus of *N. oceanica. *(A)** Effect of CLO (20 mg l^-1^), and TBF (2.5 mg l^-1^) on the maximum photosynthetic efficiency of PSII. **(B)** Transcript levels of *RBCL* gene of *N. oceanica* induced by CLO (20 mg l^-1^) or TBF (2.5 mg l^-1^). **(C)** Levels of sterols, chlorophylls, and carotenoids in the presence of CLO and TBF. Values were normalized (100% for mock control). **(D-F)** Transmission electron microscopy analysis of algal cells cultured with DMSO control **(D)**, CLO **(E),** and TBF **(F)**. **(G-H)** Scanning electron microscopy analysis of algal cells treated with DMSO control **(G)** or TBF **(H)**. Algal cells were grown in medium containing CLO, TBF, or an equivalent amount of DMSO (control) for 96 h under 50 μmol photons m^-2^ s^-1^ light. Scale bars represent 0.5 μm. Asterisks (*) indicate *P* values < 0.05.

The preceding observations provide evidence of a regulatory role of light in microalgal sterol biosynthesis and the involvement of sterol biosynthesis in photodamage at both the metabolic and gene expression levels. However, whether changes of sterol biosynthesis modulate photosynthesis is still unknown. Thus, we further probed the cellular changes that occurred in response to pharmacological perturbation of sterol biosynthesis. Cells were grown for 96 h under 50 μmol photons m^-2^ s^-1^ in the presence of 20 mg l^-1^ CLO, which inhibits DXS activity. This led to a decrease in photosynthetic efficiency (Figure 5A). RBCL is the large subunit of RuBisCO, which incorporates inorganic CO_2_ into organic forms during photosynthesis [44]. *RBCL* was transcriptionally depressed following CLO administration (Figure 5B). DXS inhibition led to reduced accumulation of sterols in particular, and of carotenoids and chlorophylls to a much lesser extent (Figure 5C). CLO-treated cells revealed less dense cytoplasm and less distinct organelles. Plastids showed a reduced number of thylakoid membrane structures and a deficiency of normal thylakoid stacking compared with the wild-type cells (Figure 5D,E). This likely explains the decreased photosynthetic efficiency of these cells. Although we showed that sterols were significantly reduced by CLO treatment, determining the contribution of sterols to photosynthetic function required the use of a more specific inhibitor. Inhibition of the post-squalene sterol biosynthetic pathway by TBF led to a 24% drop in photosynthetic efficiency relative to the control within 96 h (Figure 5A). Meanwhile, *RBCL* was transcriptionally decreased (Figure 5B). TBF-treated cells also contained fewer sterols and more carotenoids and had slightly reduced chlorophyll content (Figure 5C). The cells displayed an aberrant membrane structure with severely affected chloroplasts (Figure 5F). In addition, TBF-treated cells were misshapen compared to untreated cells (Figure 5G,H), implying a defect in membrane structure and function. Therefore, sterols are apparently required for membrane structure, including those of the chloroplast, and this is manifested in reduced photosynthetic function when sterol biosynthesis is inhibited.

### Chemical genetic analysis reveals feedback regulation of sterol biosynthesis in *N. oceanica*

In the *N. oceanica* IMET1 genome, genes for a full MEP pathway for the production of IPP as the building block for isoprenoids were identified (Figure 1). For the MVA pathway, a gene encoding hydroxy-methyl-glutaryl-CoA synthase (HMGS, g249), the first committed enzyme in the MVA pathway, was identified; however, genes encoding enzymes of the remaining steps (including the key regulatory enzyme hydroxy-methyl-glutaryl-CoA reductase; HMGR) were apparently absent (Figure 1). Since the MEP pathway is the sole source of isoprenoids, including sterols, in *N. oceanica*, the control of sterol biosynthesis in the context of IPP biosynthesis is of physiological importance. In animals, cholesterol biosynthesis is highly regulated. Cholesterol over-accumulation leads to decreased biosynthesis of cholesterol and FA, whereas low cholesterol stimulates their synthesis. HMGR, the committed enzyme in isoprenoid and sterol biosynthesis, serves as the primary feedback regulation site to ensure maintenance of lipid homeostasis [45]. Although animal and fungal HMGR is the key target for such regulation, and all such organisms employ an HMGR-binding protein called ‘Insig’, the signals and molecular mechanisms vary across these species [46]. Higher plants use both MEP and MVA pathways for isoprenoid biosynthesis. Within plant cells, IPP can be exchanged between the cytosol and plastids. Depletion of endogenous sterols by TBF triggers a significant increase in HMGR enzyme activity [34], implying a feedback mechanism in plants similar to that of mammals or fungi. However, the possible regulation of *DXS* in such a positive feedback regulation is still poorly understood. In higher plants the contribution of IPP from the MVA pathway might attenuate any feedback regulation caused by sterol depletion because of crosstalk between the MEP and MVA pathways. As a result, *N. oceanica*, which only possesses the MEP pathway, can be an ideal research model for the regulatory role of sterols with respect to *DXS*. Therefore, we investigated the transcriptional changes of *DXS* in *N. oceanica* cells in response to sterol depletion caused by CLO and TBF treatments, as well as the cellular response to adding cholesterol to the growth medium.

**Figure 6:**
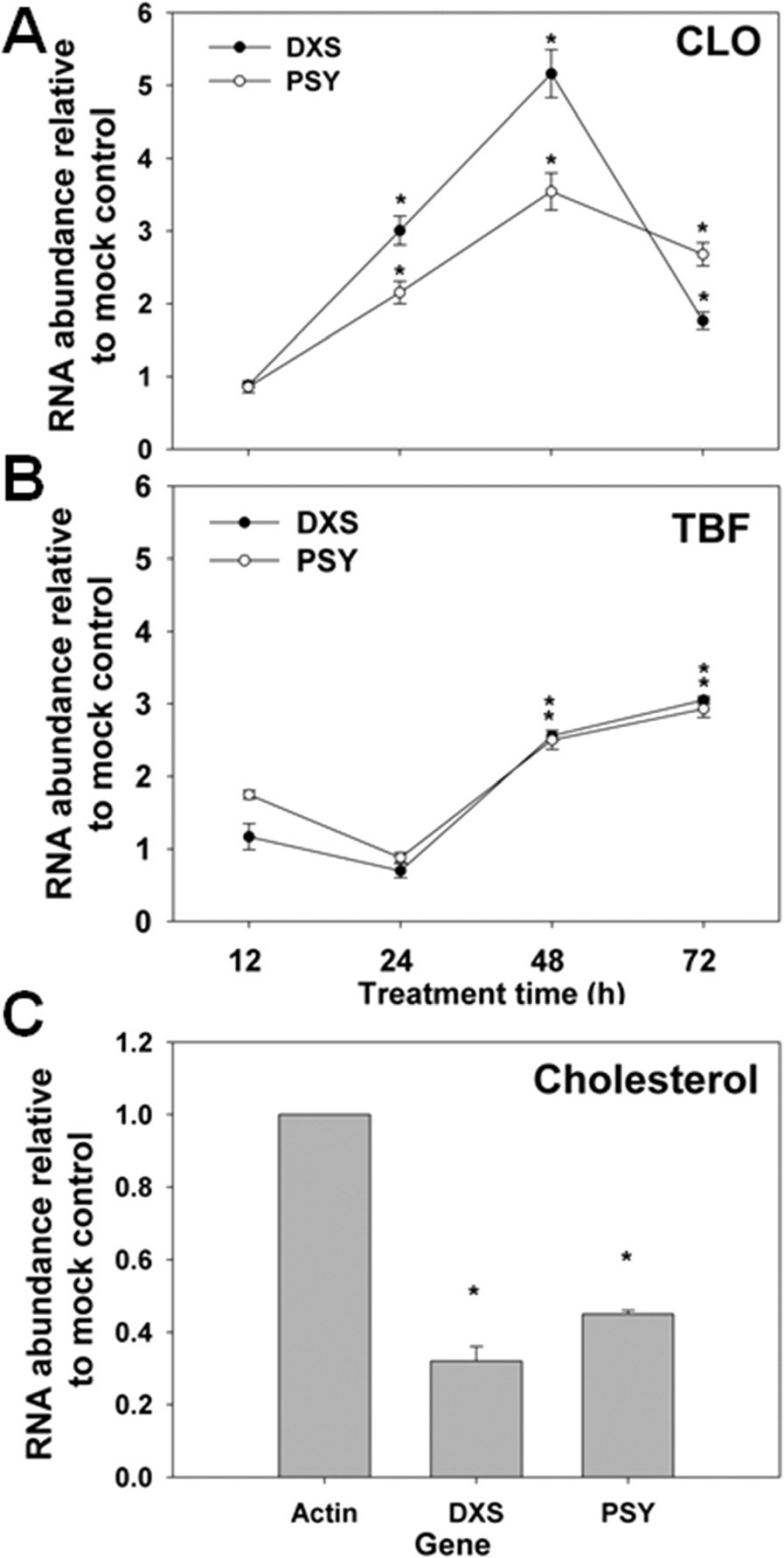
**Feedback regulation of sterol biosynthesis at the point of *DXS *gene. (A)** Transcript levels of *DXS* and *PSY* genes of *N. oceanica* induced by CLO (20 mg l^-1^). **(B)** Transcript levels of *DXS* and *PSY* genes in *N. oceanica* induced by TBF (2.5 mg l^-1^). **(C)** Transcript levels of *DXS* and *PSY* genes relative to actin gene (g3056) in response to cholesterol treatment (1.1 mg l^-1^). Samples cultured with DMSO are set as mock controls. Values are normalized to 1. Asterisks (*) indicate *P* values < 0.05.

*DXS* transcript abundance increased markedly within 48 h following CLO administration and then decreased, which is a typical inhibition-adaptation response (Figure 6A). The total sterol level was reduced to about 76% relative to the control after 96 h of CLO treatment, which can be explained by the reduced supply of IPP due to inhibition of DXS activity (Figure 5C). Moreover, levels of carotenoids and chlorophylls also decreased (Figure 5C), further confirming the low activity of isoprenoid biosynthesis and the committed role of DXS in isoprenoid biosynthesis.

**Figure 7:**
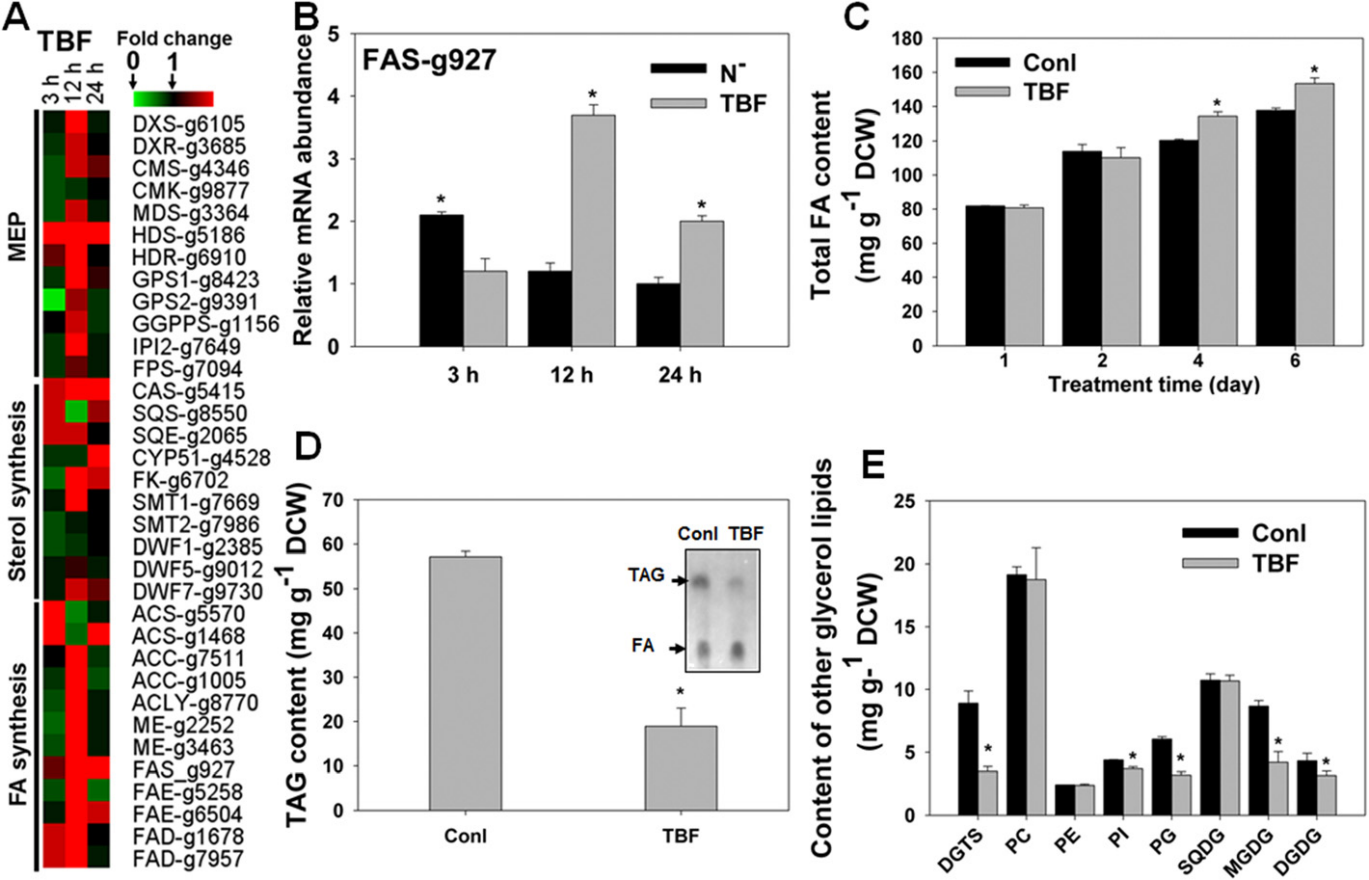
**Changes in transcripts and lipids of *N. oceanica *in response to sterol starvation induced by TBF. (A)** Transcript profiles of cholesterogenic genes and fatty acid (FA) biosynthetic genes in response to TBF (2.5 mg l^-1^). Red and green indicate up- and down-regulated genes, respectively. **(B)** Transcript levels of *FAS-*g927 gene in *N. oceanica* induced by TBF (2.5 mg l^-1^) and nitrogen depletion (N^-^). **(C)** Changes in total FA content induced by TBF. **(D)** Changes of total TAG content induced by TBF. **(E)** Changes of glycerol lipid classes (other than TAG) induced by TBF. For all experiments, cultures in exponential phase were treated with TBF (2.5 mg l^-1^) or an equivalent amount of DMSO (control). FA content was analyzed at two, four and six days, and glycerolipid content was analyzed at six days. Asterisks (*) indicate *P* values < 0.05.

Following TBF application, the total sterol content declined about 20% (Figure 5C), whereas *DXS* was transcriptionally induced after 48 h of treatment (Figure 6B). The induction cannot be explained by the depletion of IPP, since a high level of squalene accumulated in TBF-treated cells (Table 1), suggesting a sufficient supply of IPP for triterpene synthesis. Furthermore, *SQS* mRNA was reduced to 30% after 12 h of treatment (Figure 7A), a result that could potentially be caused by product inhibition by squalene accumulation. On the other hand, carotenoid levels were elevated by 40% (Figure 5B), and the phytoene synthase gene (*PSY*) encoding the key committed enzyme step for carotenoid biosynthesis was also transcriptionally up-regulated (Figure 6B). These observations point to stimulated isoprenoid biosynthesis that may be caused by sterol depletion. In addition, the entire MEP pathway and committed cholesterogenic genes were transcriptionally induced by TBF (Figure 7A). Together, the results suggest that TBF-induced sterol starvation stimulates the transcription of *DXS* and sterol biosynthetic genes.

In the case of sterol accumulation achieved by exogenously added cholesterol, the *DXS* transcript level was markedly reduced (Figure 6C). Although the carotenoid content did not change, *PSY* was transcriptionally reduced, supporting the proposal that sterol can act as a feedback regulator to the overall isoprenoid biosynthesis pathway. In summary, the transcription of *DXS* may be controlled by sterol levels, and sterol biosynthesis potentially exerts feedback regulation on isoprenoid biosynthesis, including cholesterogenesis.

### Homeostasis between sterols and lipids in *N. oceanica*

Sterol and FA biosynthesis are two major lipid synthetic pathways in eukaryotes, but it is not clear whether they are co-regulated in microalgae. In animals, type I fatty acid synthase (FAS) and other biosynthetic genes are regulated by cellular sterol levels, as are genes that encode important proteins of cholesterol metabolism [13]. To test the link between sterol and FA biosynthesis in *N. oceanica*, the transcript abundances of lipid metabolism genes were investigated following TBF-induced sterol depletion. TBF triggered an increase in type I *FAS* (g927) transcription after 12 and 24 h treatment (Figure 7A). This gene was also transcriptionally elevated in cells treated with nitrogen depletion, which is a critical trigger for lipid accumulation (Figure 7B). Consistent with this, the FA level increased over a period of several days following TBF administration (Figure 7C). Next, we analyzed the TAG content after six days of TBF treatment and observed that the TAG concentration was greatly reduced compared to that of the untreated controls (Figure 7D). Therefore, inhibition of sterol biosynthesis by TBF results in an increase in FAs, but these are not accumulated in TAG. This result is supported by thin layer chromatography of FAs (Figure 7D, inset). Simultaneously, the membrane glycerolipids, particularly the photosynthetic membrane lipids such as monogalactosyldiacylglycerol (MGDG), digalactosyldiacylglycerol (DGDG), and phosphatidylglycerol (PG), decreased significantly following TBF treatment (Figure 7E), which can explain the deformed chloroplast and the decreased photosynthetic efficiency of TBF-treated algal cells. Moreover, a remarkable reduction of phosphatidylinositol (PI) and diacylglyceroltrimethylhomoserine (DGTS) was observed (Figure 7E). Taken together, the evidence suggested that the elevated level of total FA in TBF inhibited cells was derived from the increase in *de novo* FA biosynthesis other than that of storage lipids or membrane lipids. Moreover, free FA should represent a large proportion of the increased amount of total FA.

We therefore propose a feedback system in *Nannochloropsis* for the regulation of both sterol and FA homeostasis, which is characterized by (*i*) the induction of sterol and FA biosynthesis by sterol depletion or the inhibition by sterol accumulation and (*ii*) transcriptional feedback regulation of *DXS* ensuring maintenance of lipid homeostasis. Interestingly, in animals, HMGR of the MVA pathway is the committed enzyme in isoprenoid and sterol biosynthesis, and serves as the primary site of the feedback regulation [45], suggesting similarity between the algal system and the animal model [17].

## Discussion

### Phylogenetic diversity of sterol biosynthetic pathways

Genomic comparison of evolutionarily tractable algae (Additional file 3: Table S2) revealed the presence of *HMGS* genes in all of the sampled algal genomes (except *Micromonas* sp. RCC299 and *Ostreococcus tauri* which underwent genomic reduction; [47,48]). (See Figure 1 and Additional file 1). Phylogenetic analysis of HMGS sequences suggests that eukaryotes may have inherited their *HMGS* genes from Archaea (Additional file 2: Figure S6a). Moreover, purifying selection is apparent, as the nonsynonymous substitution rate is significantly lower than the synonymous substitution rate (Additional file 3: Table S3). On the other hand, *HMGS* was actively transcribed and regulated in *N. oceanica* under TBF-induced cholesterol depletion (Additional file 2: Figure S6b). In addition, HMGSs from *N. oceanica* and *Ectocarpus siliculosus* harbor not only the functional domain of HMGS (PF01154 and PF08540) but also that of beta-ketoacyl synthase (PF00109); see Additional file 3: Table S4. As all remaining enzymes in the MVA pathway are absent in *N. oceanica*, HMGS likely performs a function unrelated to the MVA pathway in this microalga. However, an intermediate form (having four of the five key enzymes of the MVA pathway) was identified in *P. tricornutum* (Figure 1). Thus, the MVA pathways seem phylogenetically diverse among algal species.

*N. oceanica* produces phytosterols as minor products and cholesterol as the major product; such a sterol profile carries features of both animals and higher plants. Cholesterol and related sterols (ergosterol in yeast and phytosterols in plants) are considered a hallmark of eukaryotes and may even have triggered the evolution of multicellular organisms [49]. In higher plants, cholesterol is normally a minor component of the overall sterol profile (1% to 5%), and high cholesterol content may have adverse effects on plant growth [50,51]. In some plants, preference in the subcellular distribution of cholesterol was observed, being particularly enriched in chloroplasts and mitochondria and accounting for 24% of total sterol in each of the organelles (versus 1% in *Phaseolus vulgaris* leaves) [52]. *N. oceanica*, with an extremely high cholesterol content (>70%), adds another layer of diversity of sterol biosynthesis and should be an ideal model for studying the functional relationship between cholesterol and photosynthesis.

Moreover, the architecture of the *N. oceanica* sterol biosynthetic pathway also displays both animal and plant features. The underlying mechanisms remained elusive, but evolutionary pressure may have driven the production or elimination of certain sterol structures to adapt to a particular niche. Yeast strains carrying mutations early in the ergosterol pathway exhibit lower survival rates than those with mutations at later parts of the pathway [53]. An *SMT2*-overexpressing *Arabidopsis* mutant exhibited an altered campesterol-to-sitosterol ratio and dramatic phenotypic alterations, whereas a *CYP710A1*-overexpressing mutant, with an equivalent campesterol-to-stigmasterol ratio, carried a normal phenotype [54]. These results suggest a crucial role of the order of enzymes in the sterol biosynthetic pathway. In addition, *N. oceanica* harbors an animal-signature DHCR24 instead of a plant-type DWF1. Besides the roles in sterol and BR synthesis, DWF1 has a Ca^2+^/calmodulin domain related to BR signaling [55]. The domain is absent in animal DHCR24, implying that *N. oceanica* uses a different BR signaling cascade from that of higher plants. Therefore, although the backbone of the sterol biosynthetic pathway appears to be highly conserved in eukaryotic cells, variations in sterol profile and pathway architecture likely have contributed to the distinct morphology.

### Active involvement of sterol metabolism in *N. oceanica* growth and photosynthesis

The profile of sterols and the transcriptional activity of their biosynthetic genes exhibit dynamic patterns during cell proliferation. Sterols are abundantly synthesized during the late stages of algal cell proliferation, coincident with a period of rapid cell division and membrane synthesis. Defects in the proliferation of cells whose *CPI1*, *CYP51*, *SQS*, and *SMT* were blocked by specific chemical inhibitors may result from membrane structural defects due to a deficiency in specific sterols. In *Arabidopsis*, morphological defects in *hmg*[56], *cyp51*[3], and *cas1*[2] mutants were shown to be associated with cell wall gaps and aberrant cell wall thickenings. An *SMT2*-overexpressing *Arabidopsis* plant, with a higher ratio of campesterol to sitosterol, displayed pleiotropic effects on development, such as reduced growth, increased branching, and low fertility [54]. In addition, sterols transcriptionally activate genes associated with cell expansion and proliferation [12]. Sterol biosynthetic genes are strongly induced at the late-log stage of *N. oceanica*. The inhibition of cell proliferation by the chemical blockage of *CPI1*, *CYP51*, *SQS*, or *SMT* may be due to defects in sterol biosynthesis and composition.

The inhibition of DXS (responsible for IPP biosynthesis including sterol biosynthesis) and SQE is associated with aberrant photosynthetic efficiency in *N. oceanica*. The defective photosynthesis of DXS-blocked cells may be due to the joint effects of the reduced levels of sterols, carotenoids, and chlorophylls which impair membrane stability as well as light-absorption capacity and management of reactive oxygen species. In contrast, only sterol synthesis is affected in SQE-blocked cells, precluding the possibility that the damage to photosynthesis is caused by a defect of other isoprenoids. Both CLO- and TBF-treated *N. oceanica* cells display a deformed shape. This appeared to suggest that some of the physiological effects observed were the indirect result of the chemical inhibitor (for example, caused by the well-described toxicity of an increasing pool of free FA in TBF-treated cells). However, specificities of these chemical inhibitors have been confirmed [34-38]; moreover, free FA levels altered in neither CLO-treated (over the full time course of treatment) nor TBF-treated cells (during the first 24 h; despite the decrease of photosynthetic efficiency in the same time frame). It is therefore unlikely that the cell deformation is the only cause for the observed physiological differences, particularly photosynthesis. Furthermore, whereas sterol biosynthetic genes were transcriptionally induced by light, the biosynthesis was reduced upon high light in *N. oceanica*. On the other hand, transcription of *SMT* was up-regulated upon high light stress in *C. reinhardtii*[57]. Therefore, sterols play an active role in the response to high light stress in microalgae; moreover, changes in the relative abundance of specific sterols are associated with distinct abnormalities in photosynthesis.

### Feedback regulation of sterol biosynthesis and role of sterols in lipid homeostasis

The phytosterol biosynthetic pathway leads to production of BRs, which were previously the only known steroid signaling molecules in plants [58]. However, later studies suggest that other sterols besides BRs also function as signaling components, regulating development and gene expression in plants in a manner analogous to the action of cholesterol in mammalian systems [12]. We demonstrated that sterols may also act as signaling molecules in microalgae. In this system, *DXS* possesses a feedback regulation mechanism in response to sterol starvation. The inhibition of SQE leads to a nearly 20% decrease of the sterol level, a pronounced inhibitor-mediated increase of the carotenoid level and a remarkable concomitant transcriptional increase of cholesterogenic transcripts. In humans (and other mammals), *de novo* synthesis of FAs requires cholesterol-mediated regulation of the type I *FAS* promoter, suggesting that lipogenesis and sterol synthesis are coordinately regulated by cholesterol [59]. There are many genes contributing to FA synthesis, among which type I *FAS* is one of the most intensively studied. Whereas type I *FAS* genes appear to be absent in the *C. reinhardtii* genome, an *N. oceanica* IMET1 type I *FAS* (g927) was transcriptionally up-regulated during triacylglycerol biosynthesis under nitrogen-depletion conditions, suggesting that type I *FAS* genes may contribute additional FA for the synthesis of TAG in *N. oceanica*[23]. This type I *FAS* gene was also significantly elevated during the induced depletion of sterol in *N. oceanica*. Moreover, in *N. oceanica*, during the induced depletion of sterol, the free FA level was stimulated, despite the unknown response of the other FA biosynthetic genes (such as type II *FAS* genes; [23,25]*.* Thus, sterols likely control the homeostasis of cholesterol and FA biosynthesis in *N. oceanica*. If so, this would suggest an ancient emergence of the co-regulation of the biosynthesis of FAs and sterols.

## Conclusion

In summary, in microalgae, sterols play essential roles in growth, photosynthesis, and adaption to high light stress. Sterol biosynthesis is potentially feedback-regulated at node of DXS. Sterol and FA metabolisms are co-regulated, such that sterols promote the incorporation of FAs into TAG. Understanding these regulatory mechanisms may lead to rational strategies for improved growth characteristics for *N. oceanica* under challenging environmental conditions and for the development of improved microalgal feedstocks for high-value products or biofuel.

## Materials and methods

### Algal culture

*N. oceanica* IMET1 was inoculated into modified f/2 liquid medium, which was prepared with 35 g l^-1^ sea salt, 1 g l^-1^ NaNO_3_, 67 mg l^-1^ NaH_2_PO_4_*H_2_O, 3.65 mg l^-1^ FeCl_3_*6H_2_O, 4.37 mg l^-1^ Na_2_EDTA*2H_2_O, trace metal mix (0.0196 mg l^-1^ CuSO_4_*5H_2_O, 0.0126 mg l^-1^ NaMoO_4_*2H_2_O, 0.044 mg l^-1^ ZnSO_4_*7H_2_O, 0.01 mg l^-1^ CoCl_2_, and 0.36 mg l^-1^ MnCl_2_*4H_2_O), and vitamin mix (2.5 μg l^-1^ VB_12_, 2.5 μg l^-1^ biotin, and 0.5 μg l^-1^ thiamine HCl). The cells were grown in liquid cultures under continuous light (approximately 50 μmol photons m^-2^ s^-1^) at 25°C [23].

Algal cells at certain growth stages were collected for sterol profiling and transcript analysis. Mid-logarithmic phase algal cells were collected and washed with axenic seawater. Equal numbers of cells were re-inoculated in nitrogen-replete medium and nitrogen-deprived medium with 50 μmol m^-2^ s^-1^ light intensity. Cell aliquots were collected for RNA isolation after being transferred to the designated conditions for 3, 12, and 24 h. Three biological replicates of algal cultures were established for each treatment [23].

Total RNA isolation and quantitative real-time PCR (qPCR) analysis were carried out as previously described [60]. The relative amounts were normalized to the respective beta-actin transcripts. The 2^-ΔΔCT^ method was used to analyze qPCR data. Each value in the graphs shows the mean with SE of three experiments. The primer sequences for each gene are given in Additional file 3: Table S5.

### Data sources, retrieval, and phylogenetic analyses of the sterol biosynthetic enzymes in algae

The *Arabidopsis* protein sequences were retrieved from http://www.arabidopsis.org/. Microalgal genomes were retrieved from the links in Additional file 3: Table S2. Proteins from all genomes were blasted with the *Arabidopsis* protein queries (Additional file 3: Table S1) using the standalone BLAST software package. The Conserved Domain Database (http://www.ncbi.nlm.nih.gov/cdd/), Pfam (http://pfam.sanger.ac.uk/search), and SMART [61] plus previous genome annotations were employed to confirm the accuracy of chosen sequences. If more than one allele existed, the best-match allele was chosen as representative for the similarity heatmap analysis. The sequences ultimately selected are listed in Additional file 1. Gaps and ambiguously aligned sites were removed using CLUSTALW [62], gBlock [63], and manual validation. ProtTest [64] and PhyML 3.0 [65] were used to select the available model of protein substitution and phylogenetic analyses with a maximum likelihood method. Bootstrap support values were estimated using 100 pseudo-replicates.

### Lipid analysis

Lipids were extracted from lyophilized material according to the method by Yoon [66]. Sterol profiles were analyzed according to our previous description [35]. Lipid class analyses were used according to thin layer chromatography (TLC) and quantified using the gas chromatography-fatty acid methyl esters (GC-FAME) method as previously described [66].

### Chemical treatments

Cultures in exponential phase were diluted in fresh media and grown for 12 h. Subsequently, cultures were added with inhibitors at a gradient concentration, or an equivalent amount of DMSO (not to exceed a final concentration of 0.1%). Cholesterol solution (10 mg ml^-1^, acetone) was mixed with Tween-20 acetone solution to the designed concentration, and the acetone was removed by a stream of nitrogen. The residue was reconstructed to a water solution and rigid vortex. The solution was sterilized via filtration by passing it through a 0.25-μm filter, and it was then added to the culture medium to a desired concentration. Tween-20 without sterol was treated as a blank control. Samples were taken at the indicated periods of time for further analysis.

### Analysis of sterols and transcripts related to sterol metabolism

Cells at mid-log phase were: (*i*) grown under constant light intensities 100 and 300 μmol photons m^-2^ s^-1^. The 96 h old cells were taken for transcript and sterol profiling analysis; (*ii*) transferred to darkness for 12 h and then transferred to 300 or 100 μmol photons m^-2^ s^-1^; and samples were collected after 12 h; (*iii*) cultivated under 300 μmol photons m^-2^ s^-1^ for 12 h, and then transferred to 100 μmol photons m^-2^ s^-1^; samples were collected after 12 and 24 h; and (*iv*) cultivated under 100 μmol photons m^-2^ s^-1^ for 12 h, and then transferred to 50 μmol photons m^-2^ s^-1^; samples were collected after 12 and 24 h.

### Microscopy

Four-day-old *N. oceanica* cells grown in medium containing CLO (20 mg L^-1^), TBF (2.5 mg L^-1^), or an equivalent amount of DMSO were sampled for transmission electron microscopy (TEM) [67] and scanning electron microscopy (SEM) analysis [68].

### Statistical analysis

All experiments were replicated at least three times. Data were analyzed using ANOVA followed by paired or unpaired Student’s *t* tests. Differences with *P* < 0.05 were considered significant. Asterisks (*) indicate *P* values < 0.05.

## Abbreviations

25-AZA: 25-azalanosterol; BR: brassinosteroid; CAS: cycloartenol synthase; CLO: clomazone; CPI: cycloeucalenol cycloisomerase; CYP51: sterol 14-alpha demethylase; CYP710A: sterol C-22 desaturase; DGDG: digalactosyldiacylglycerol; DGTS: diacylglyceroltrimethylhomoserine; DHCR24: dihydrocholesterol reductase; DWF1: sterol C-24(28) isomerase-reductase; DWF5: sterol C-7 reductase; DWF7: delta7 sterol C-5 desaturase; DXS: 1-deoxy-D-xylulose 5-phosphate synthase; ERG4: sterol delta 24(28) reductase; FA: fatty acid; FAS: fatty acid synthase; FK: sterol C-14 reductase; GC-FAME: gas chromatography-fatty acid methyl esters; HMGR: hydroxy-methyl-glutaryl-CoA reductase; HMGS: hydroxy-methyl-glutaryl-CoA synthase; HYD: sterol C-8 isomerase; IPP: isopentenyl pyrophosphate; *Kn*: nonsynonymous substitutions; *Ks*: synonymous substitutions; LAS: lanosterol synthase; MEP: methyl erythritol phosphate; MGDG: monogalactosyldiacylglycerol; MVA: mevalonic acid; PG: phosphatidyl glycerol; PI: phosphatidylinositol; PSY: phytoene synthase; qPCR: quantitative real-time PCR; SBI: sterol biosynthetic inhibitor; SEM: scanning electron microscopy; SMO: sterol 4-methyl oxidase; SMT: sterol methyltransferase; SQE: squalene epoxidase; SREBP: sterol regulatory element binding protein; TAG: triacylglycerol; TBF: terbinafine; TDM: tridemorph; TEB: tebuconazole; TEM: transmission electron microscopy; TLC: thin layer chromatography.

## Competing interests

The authors declare that they have no competing interests.

## Authors’ contributions

YL and WZ planned the experiments. YL, WZ, WL, JL, JJ, and FL performed the experiments. YL and WZ analyzed the data. YL, WZ, SS, and JX wrote the paper. All authors read and approved the final manuscript.

## Supplementary Material

Additional file 1 **Dataset S1.** List of proteins involved in the sterol biosynthetic pathway used to create the similarity heatmap. Click here for file

Additional file 2: Figure S1 Phylogenetic relationship of organisms studied here. **Figure S2.** Phylogenetic analysis of the sterol 24(25) isomerase reductase (DWF1) and 24-dehydrocholesterol reductase (DHCR24) using the PhyML 3.0 program. **Figure S3.** Identification of the sterol compounds extracted from *N. oceanica*. **Figure S4.** Inhibition ratio of different isoprenoid biosynthetic inhibitors or sterol biosynthetic inhibitors. **Figure S5.** Changes in sterol biosynthesis induced by nitrogen depletion. **Figure S6.** Transcriptional dynamics and phylogenetic analysis of hydroxy-methyl-glutaryl-CoA synthases (HMGS). Click here for file

Additional file 3: Table S1 List of sterol biosynthetic enzymes in *Arabidopsis thaliana*. **Table S2.** Databases used as sequence and annotation sources. **Table S3.** The ratio of the rate of nonsynonymous substitutions (*Kn*) to the rate of synonymous substitutions (*Ks*) of hydroxy-methyl-glutaryl-CoA synthase (HMGS). **Table S4.** Comparison of sequence identity and conserved domains of hydroxy-methyl-glutaryl-CoA synthase (HMGS) proteins between algal species and *Arabidopsis thaliana*. **Table S5.** Primers used in this study. Click here for file
