# Supplementary material for: Regulation of the cholesterol biosynthetic pathway and its integration with fatty acid biosynthesis in the oleaginous microalga Nannochloropsis oceanica
Source: Biotechnol Biofuels. 2014 May 30;7:81. doi: 10.1186/1754-6834-7-81 (PMC4052811; doi:10.1186/1754-6834-7-81)
Supplement: Additional file 2: Figure S1 — Phylogenetic relationship of organisms studied here. Figure S2. Phylogenetic analysis of the sterol 24(25) isomerase reductase (DWF1) and 24-dehydrocholesterol reductase (DHCR24) using the PhyML 3.0 program. Figure S3. Identification of the sterol compounds extracted from N. oceanica. Figure S4. Inhibition ratio of different isoprenoid biosynthetic inhibitors or sterol biosynthetic inhibitors. Figure S5. Changes in sterol biosynthesis induced by nitrogen depletion. Figure S6. Transcriptional dynamics and phylogenetic analysis of hydroxy-methyl-glutaryl-CoA synthases (HMGS). [file 1754-6834-7-81-S2.pdf]

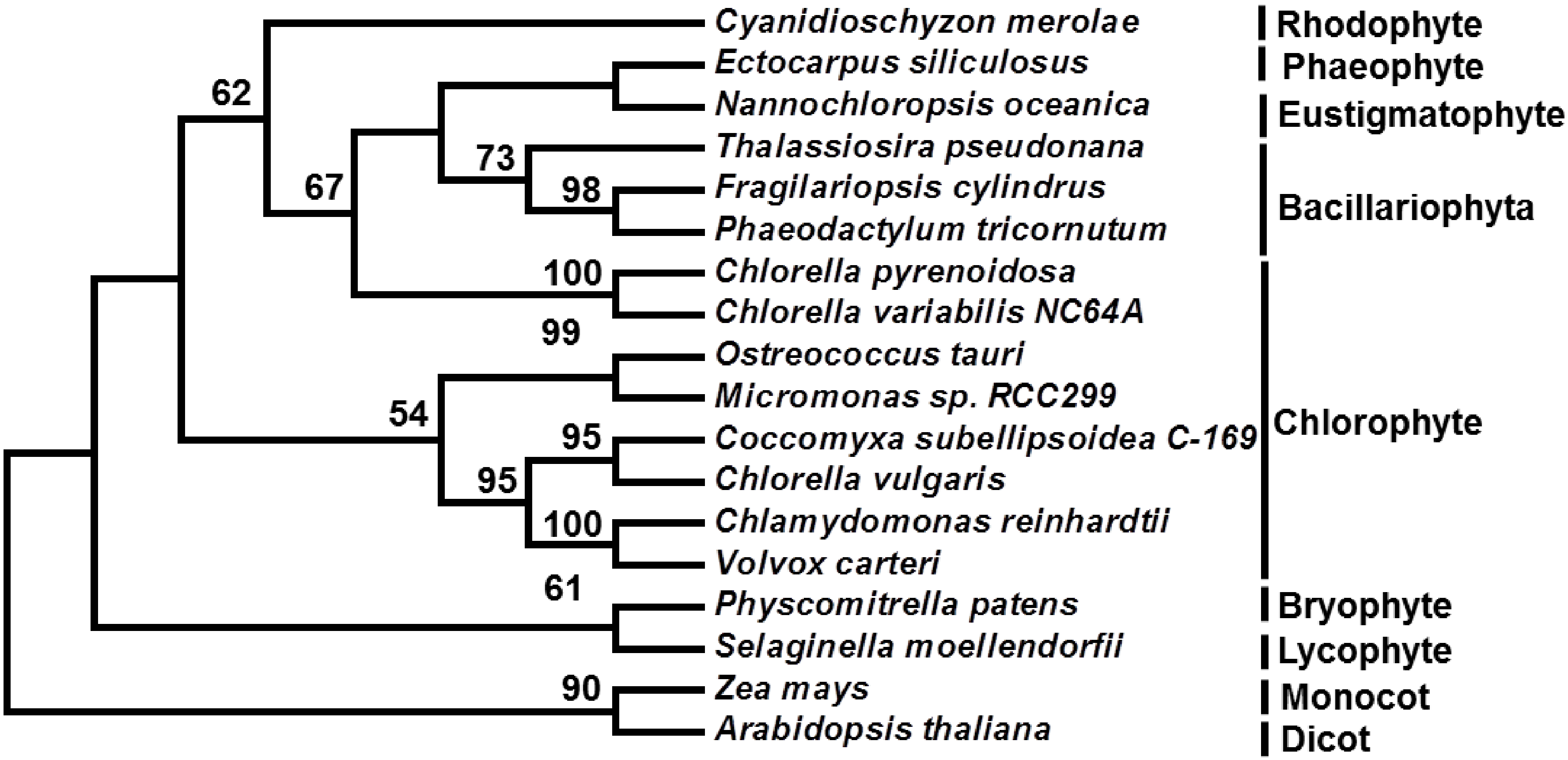

Figure S1. Phylogenetic relationship of organisms studied here.

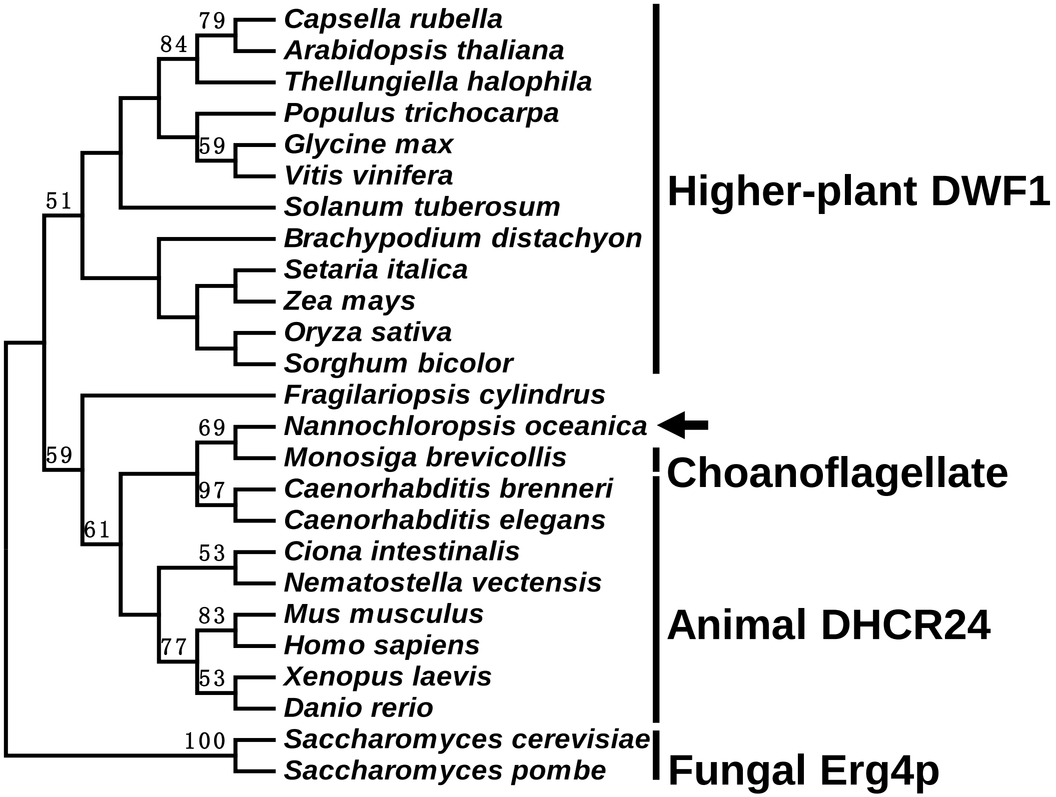

Figure S2. Phylogenetic analysis of the sterol 24(25) isomerase reductase (DWF1) and 24-dehydrocholesterol reductase (DHCR24) using the Phy ML 3.0 program. Protein identity and conserved domains of proteins used to build the tree are listed in Data S1.

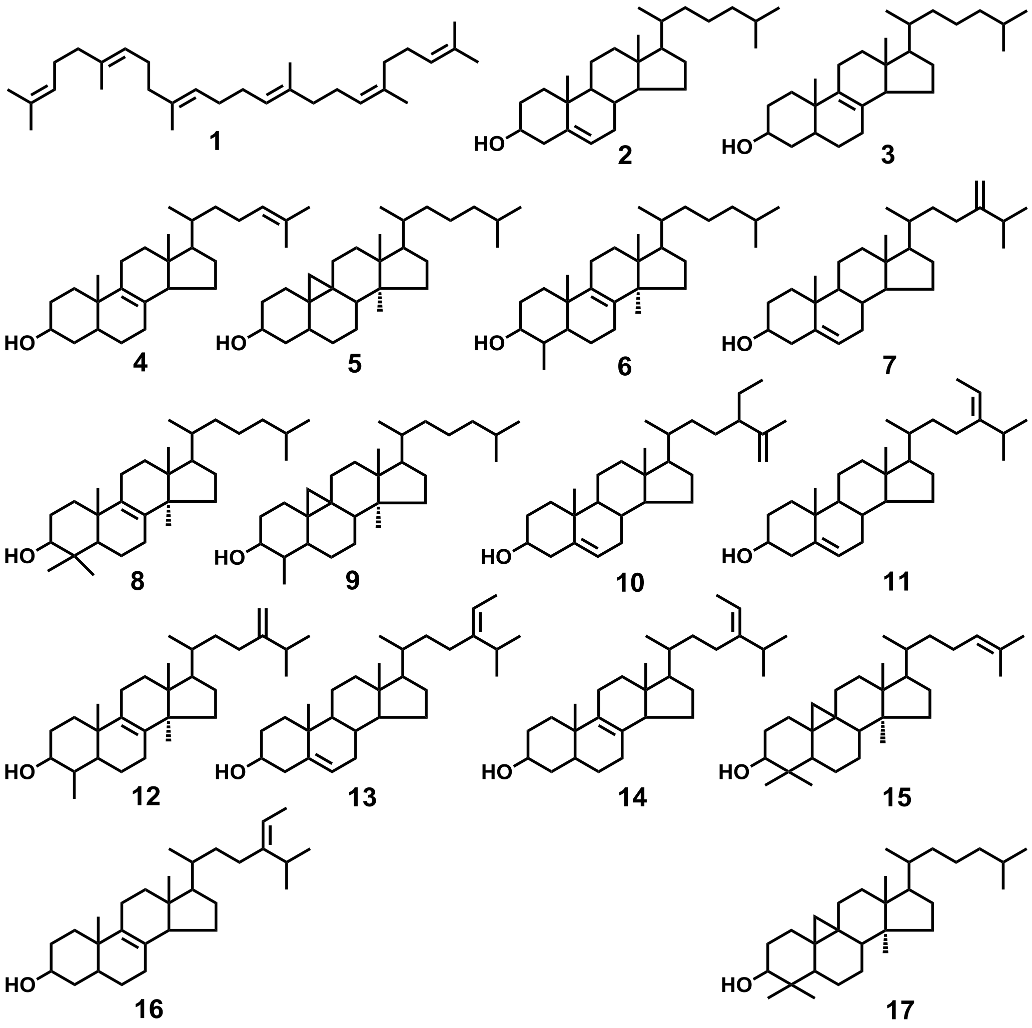

Figure S3. Identification of the sterol compounds extracted from *N. oceanica*. 1: squalene, 2: cholesterol, 3: cholest-8-enol, 4: desmosterol, 5: pollinastanol, 6: 4,14-dimethylcholest-8-enol, 7: 24-methylenecholesterol, 8: 24(25)-dihydrolanosterol, 9: 4-methylpollinasterol, 10: 24-methylcholesta-5, 25(27)-dienol, 11: fucosterol, 12: obtusifoliol, 13: isofucosterol, 14: stigmasta-8, 24(28) *E*-dienol, 15: cycloartenol, 16: stigmasta-8, 24(28) *Z*-dienol, 17: cycloartanol.

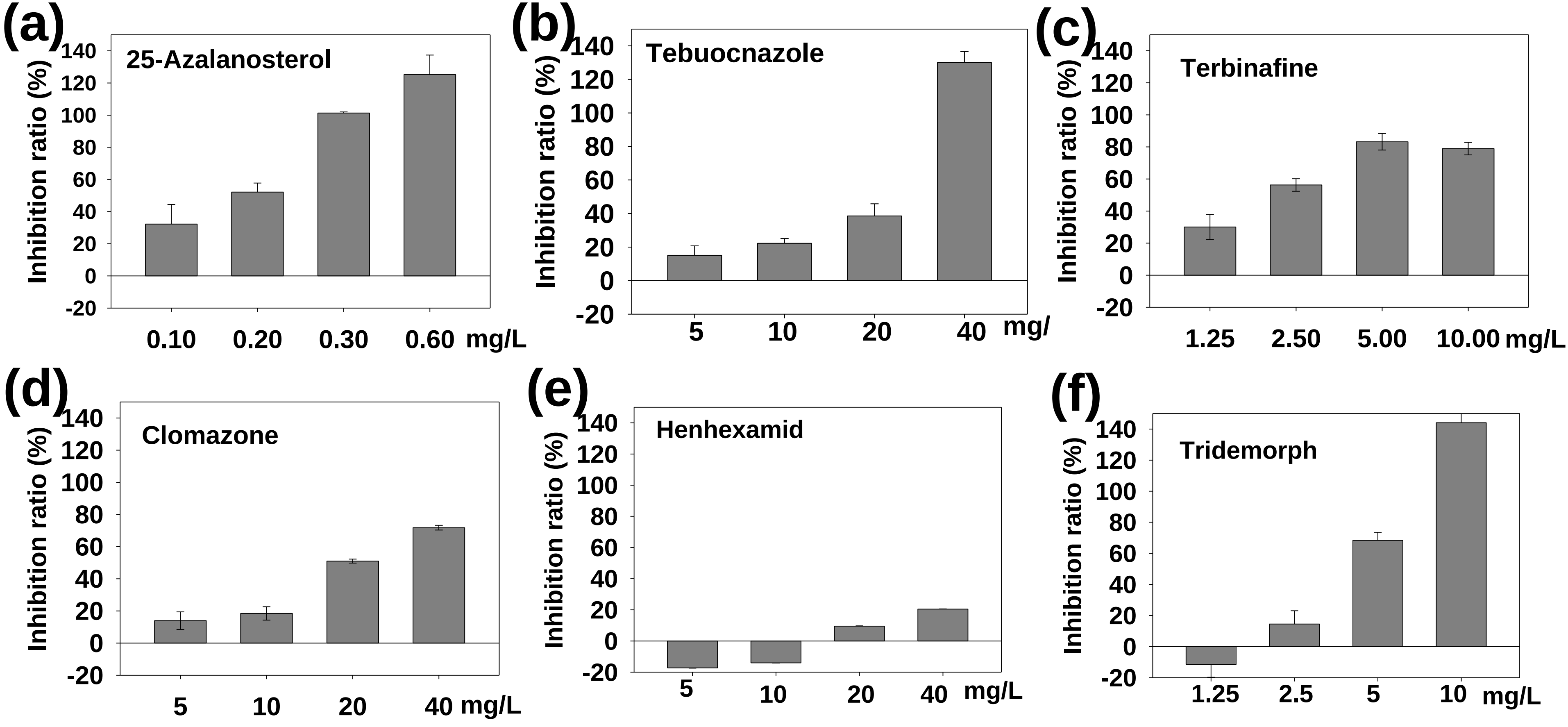

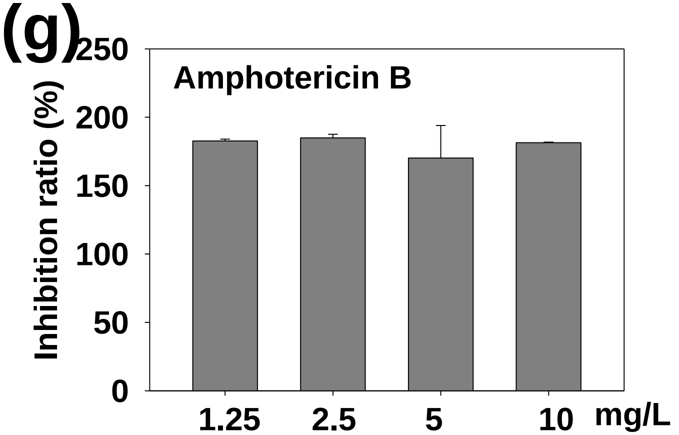

Figure S4. Inhibition ratio of different isoprenoid or sterol biosynthetic inhibitors. Culture in exponential phase was diluted in fresh medium and grown for 12 h. Subsequently, cultures were added with inhibitors at a gradient concentration, or an equivalent amount of DMSO (not to exceed a final concentration of 0.1%).

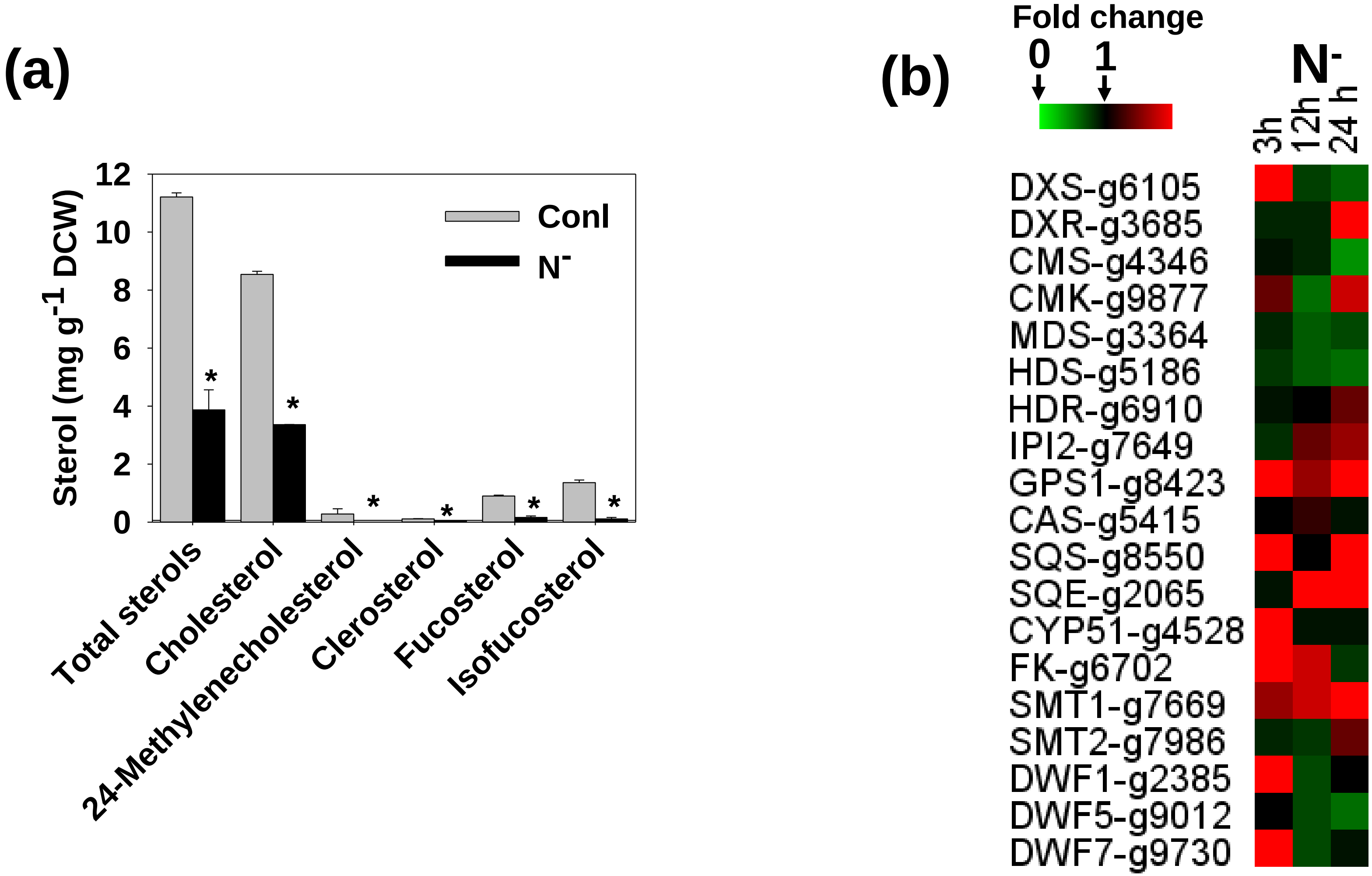

Figure S5. Changes in sterol biosynthesis induced by nitrogen depletion. (a) Changes of sterols induced by nitrogen depletion. Cells with the same initial inoculation amount were cultivated under nitrogen-replete or -deplete conditions for transcription analysis and sterol profiling (6 d). (b) Transcriptional dynamics of cholesterogenic genes of *N. oceanica* in response to nitrogen depletion. Red and green indicate up- and down-regulated genes, respectively. The time points for the cluster analysis are indicated in hours at the top. Values are the means of three replicates and are representative of a standard experiment.

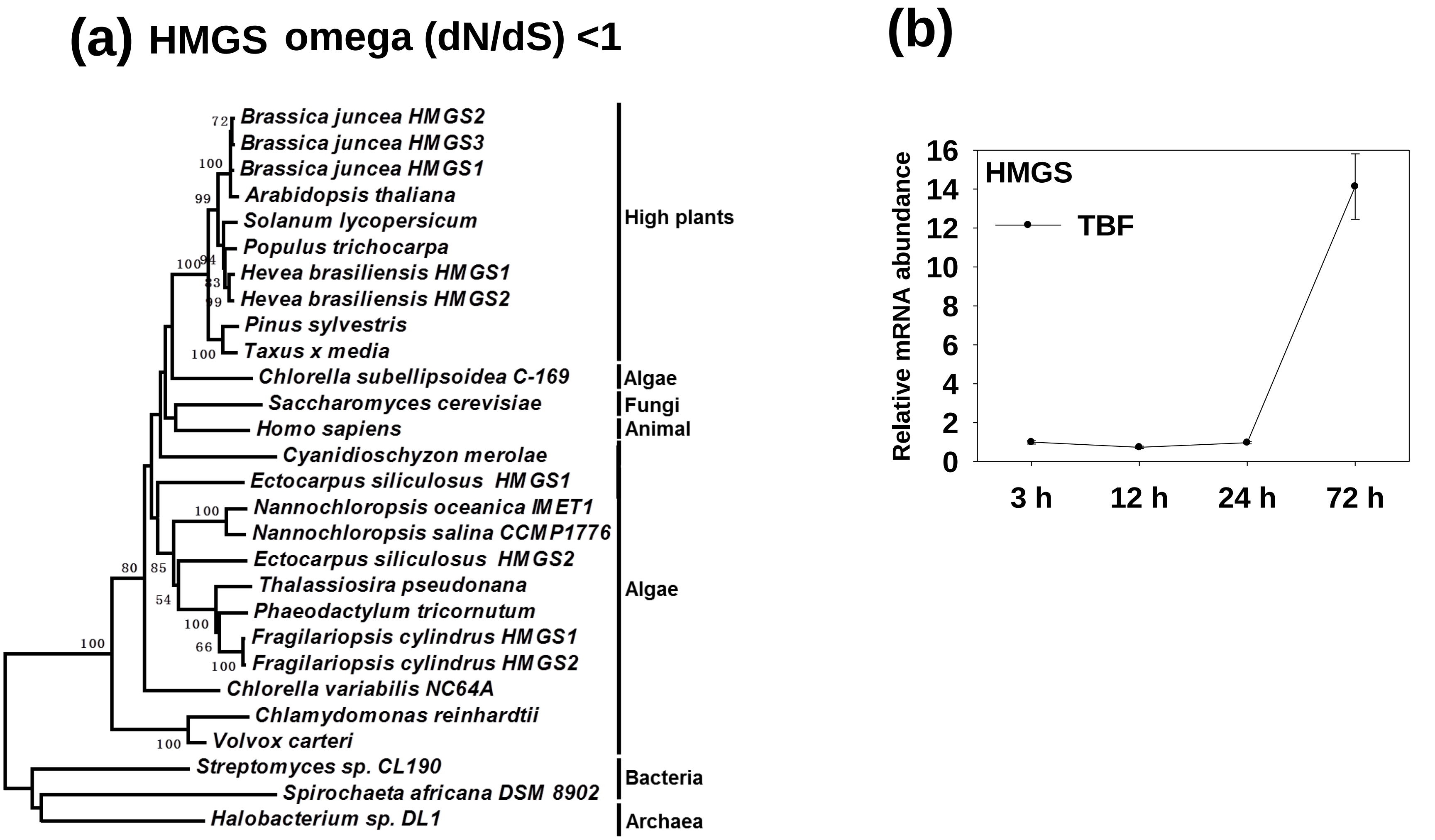

Figure S6. (a) Phylogenetic analysis of the Hydroxyl-Methyl-Glutaryl-CoA Synthase (HMGS) using Phy ML 3.0 program. Protein identity and conserved domains of proteins used to build the tree are listed in Table S3. (b) Transcriptional dynamics of *HMGS* in *N. oceanica* following TBF-induced sterol depletion.
