# Supplementary material for: Regulation of the cholesterol biosynthetic pathway and its integration with fatty acid biosynthesis in the oleaginous microalga Nannochloropsis oceanica
Source: Biotechnol Biofuels. 2014 May 30;7:81. doi: 10.1186/1754-6834-7-81 (PMC4052811; doi:10.1186/1754-6834-7-81)
Supplement: Additional file 3: Table S1 — List of sterol biosynthetic enzymes in Arabidopsis thaliana. Table S2. Databases used as sequence and annotation sources. Table S3. The ratio of the rate of nonsynonymous substitutions (Kn) to the rate of synonymous substitutions (Ks) of hydroxy-methyl-glutaryl-CoA synthase (HMGS). Table S4. Comparison of sequence identity and conserved domains of hydroxy-methyl-glutaryl-CoA synthase (HMGS) proteins between algal species and Arabidopsis thaliana. Table S5. Primers used in this study. [file 1754-6834-7-81-S3.pdf]

Table S1. Databases used as sequence and annotation sources. Species names are provided along with descriptions and database names, appropriate references and web links (URLs). We recruited representatives from five lineages of microalgae: (*i*) green algae, including the picophytoplankton *Micromonas* sp. RCC299 (Worden *et al.*, 2009); the free-living, small-genome alga *Ostreococcus tauri* (Palenik *et al.*, 2007); the virus-harboring alga *Chlorella variabilis* NC64A (Blanc *et al.*, 2010a); and the laboratory model alga *Chlamydomonas reinhardtii* (Merchant *et al.*, 2007); (*ii*) red algae, including a primitive alga with a simple cellular architecture, *Cyanidioschyzon merolae* (Matsuzaki *et al.*, 2004); (*iii*) diatoms, including the marine pennate diatom *Phaeodactylum tricornutum* (Bowler *et al.*, 2008) and the centric diatom *Thalassiosira pseudonana* (Armbrust *et al.*, 2004); (*iv*) brown algae, including the marine *Ectocarpus siliculosus* (Cock *et al.*, 2010); and (*v*) Eustigmatophyta, a diverse Stramenopile group that evolved between the brown algae and diatoms, represented by *N. oceanica*.

Species	Database	Description	URL	Reference
C. merolae	*Cyanidioschyzon merolae* genome	*Cyanidioschyzon merolae* Genome Project	http://merolae.biol.s.u-tokyo.ac.jp	(Nozaki *et al.*, 2007)
P. tricornutum	JGI	DOE Joint Genome Institute	http://genome.jgi-psf.org/Phatr2/Phatr2.home.html	(Bowler *et al.*, 2008)
T. pseudonana	JGI	DOE Joint Genome Institute	http://genome.jgi-psf.org/thaps1/thaps1.home.html	(Armbrust *et al.*, 2004)
F. cylindrus	JGI	DOE Joint Genome Institute	http://genome.jgi-psf.org/Fracy1/Fracy1.home.html	
N. oceanica	EnergyAlgaeDB	A functional genomics database for energy microalgae	http://www.bioenergychina.org:8989/	(Wang *et al.*, 2013)
E. siliculosus	Orcae	Online Resource for Community Annotation of Eukaryotes	http://bioinformatics.psb.ugent.be/orcae/	(Cock *et al.*, 2010)
O. tauri	JGI	DOE Joint Genome Institute	http://genome.jgi-psf.org/Ostta4/Ostta4.home.html	(Palenik *et al.*, 2007)
***Micromonas* sp. RCC299**	JGI	DOE Joint Genome Institute	http://genome.jgi-psf.org/MicpuN3/MicpuN3.home.html	(Worden *et al.*, 2009)
C. variabilis	JGI	DOE Joint Genome Institute	http://genome.jgi-psf.org/ChlNC64A_1/ChlNC64A_1.home.html	(Blanc *et al.*, 2010b)

**NC64A**				
***C. subellipsoidea* C-169**	JGI	DOE Joint Genome Institute	http://genome.jgi.doe.gov/Coc_C169_1/Coc_C169_1.home.html	(Blanc *et al.*, 2012)
C. reinhardtii	JGI	DOE Joint Genome Institute	http://genome.jgi-psf.org/Chlre4/Chlre4.home.html	(Merchant *et al.*, 2007)
V. carteri	Phytozome	Phytozome	http://genome.jgi-psf.org/Volca1/Volca1.home.html	(Prochnik *et al.*, 2010)
A. thaliana	TAIR	The *Arabidopsis* Information Resource	http://www.arabidopsis.org/	(Initiative, 2000; Garcia-Hernandez *et al.*, 2002)

Table S2. The ratio of the rate of nonsynonymous substitutions (*Kn*) to the rate of synonymous substitutions (*Ks*) of hydroxyl-methyl-glutaryl-CoA synthase (HMGS). A *Kn/Ks* ratio of 1 is assumed to indicate neutrality, *Kn/Ks* > 1 is a signature of positive selection at the amino-acid level, and *Kn/Ks* < 1 is indicative of purifying selection.

Species	*Kn/Ks*	*Kn*	*Ks*
Halobacterium sp. DL1	0.951	0.412	0.434
Saccharomyces cerevisiae	0.651	0.315	0.484
Cyanidioschyzon merolae	0.460	0.281	0.612
Phaeodactylum tricornutum	0.386	0.200	0.519
Thalassiosira pseudonana	0.296	0.112	0.380
Fragilariopsis cylindrus	0.185	0.136	0.735
Nannochloropsis oceanica	0.437	0.255	0.583
Nannochloropsis salina	0.537	0.231	0.430
Chlorella variabilis NC64A	0.489	0.160	0.327
Coccomyxa subellipsoidea C-169	0.453	0.150	0.332
Chlamydomonas reinhardtii	0.436	0.118	0.270
Volvox carteri	0.311	0.088	0.283
Pinus sylvestris	0.181	0.034	0.190
Arabidopsis thaliana	0.168	0.048	0.287
Solanum lycopersicum	0.119	0.042	0.353
Taxus x media	0.259	0.039	0.150
Homo sapiens	0.305	0.236	0.772

Table S3. Comparison of sequence identity and conserved domains of hydroxyl-methyl-glutaryl-CoA synthase (HMGS) proteins between algal species and *A. thaliana*. Microalgal HMGS orthologs were BLASTed with *A. thaliana* HMGS. Abbreviations: *C. merolae* (Cm), *P. tricornutum* (Pt), *T. pseudonana* (Tp), *F. cylindrus* (Fc), *N. oceanica* (No), *E. siliculosus* (Es), *Micromonas* sp. RCC299 (Mi), *O. tauri* (Ot), *C. variabilis* NC64A (Cv), *C. subellipsoidea* C-169 (Cs), *C. reinhardtii* (Cr) and *V. carteri* (Vc).

Protein ID	Max Identity	Query cover	E value	Conserved domain(s)	Pfam ID
**Cm.CMM189C**	42%	98%	8e-101	HMG_CoA_synt_N and HMG_CoA_synt_C	PF01154 and PF08540
**Tp.260768**	44%	96%	6e-116	HMG_CoA_synt_N and HMG_CoA_synt_C	PF01154 and PF08540
**Pt.16649**	43%	97%	2e-109	HMG_CoA_synt_N and HMG_CoA_synt_C	PF01154 and PF08540
**Pt.33278**	31%	46%	3e-12	HMG_CoA_synt_N	PF01154
**Fc.185767**	44%	97%	2e-104	HMG_CoA_synt_N and HMG_CoA_synt_C	PF01154 and PF08540
**Fc.199578**	43%	97%	9e-106	HMG_CoA_synt_N and HMG_CoA_synt_C	PF01154 and PF08540
**No.g249**	50%	98%	3e-121	HMG_CoA_synt_N, HMG_CoA_synt_C, ketoacyl-synt	PF01154, PF08540 and PF00109
**Ns. 4043**	56%	98%	1e-119	HMG_CoA_synt_N, HMG_CoA_synt_C, ketoacyl-synt	PF01154, PF08540 and PF00109
**Es.Esi0335_0012**	44%	95%	9e-113	HMG_CoA_synt_N and HMG_CoA_synt_C	PF01154 and PF08540
**Es.Esi0131_0082**	40%	99%	2e-107	HMG_CoA_synt_N, HMG_CoA_synt_C, ketoacyl-synt	PF01154, PF08540 and PF00109
**Cs.27385**	50%	97%	2e-134	HMG_CoA_synt_N and HMG_CoA_synt_C	PF01154 and PF08540
**Cv.138158**	41%	97%	1e-119	HMG_CoA_synt_N and HMG_CoA_synt_C	PF01154 and PF08540
**Cr.118594**	37%	84%	2e-40	HMG_CoA_synt_N and HMG_CoA_synt_C	PF01154 and PF08540
**Vc.59473**	38%	97%	7e-94	HMG_CoA_synt_N and HMG_CoA_synt_C	PF01154 and PF08540

Table S4. List of sterol biosynthetic enzymes in *A. thaliana*. Protein names, descriptions, accession numbers and references are provided.

Symbol	Description	Accession	Reference
**AACT1**	acetoacetyl-CoA thiolase	AT5G47720	(Jin *et al.*, 2012)
**AACT2**	acetoacetyl-CoA thiolase	AT5G48230	(Jin *et al.*, 2012)
**HMGS**	hydroxymethylglutaryl-CoA synthase	AT4G11820	(Montamat *et al.*, 1995)
**HMGR1**	hydroxy-3-methylglutaryl coenzyme A reductase	AT1G76490	(Enjuto *et al.*, 1994; Nieto *et al.*, 2009)
**HMGR2**	hydroxy-3-methylglutaryl coenzyme A reductase	AT2G17370	(Enjuto *et al.*, 1994; Lumbreras *et al.*, 1995)
**MVK**	mevalonate kinase	AT5G27450	(Riou *et al.*, 1994)
**PMK**	phosphomevalonate kinase	AT1G31910	(Phillips *et al.*, 2008)
**MPDC1**	mevalonate diphosphate decarboxylase	AT2G38700	(Cordier *et al.*, 1999)
**MPDC2**	mevalonate diphosphate decarboxylase	AT3G54250	(Cordier *et al.*, 1999)
**DXS1**	1-deoxy-D-xylulose 5-phosphate synthase 1	AT4G15560	(Phillips *et al.*, 2008)
**DXS2**	1-deoxy-D-xylulose 5-phosphate synthase 2	AT3G21500	(Carretero-Paulet *et al.*, 2013)
**DXS3**	1-deoxy-D-xylulose 5-phosphate synthase 3	AT5G11380	(Phillips *et al.*, 2008)
**DXR**	1-deoxy-d-xylulose 5-phosphate reductoisomerase	AT5G62790	(Lichtenthaler, 1999; Schwender *et al.*, 1999)
**CMS**	4-diphosphocytidyl-2C-methyl-D-erythritol synthase	AT2G02500	(Rohdich *et al.*, 2000)
**CMK**	4-(cytidine 5'-diphospho)-2-C-methyl-D-erythritol kinase	AT2G26930	(Hsieh & Goodman, 2005)
**MDS**	2C-methyl-D-erythritol 2,4-cyclodiphosphate synthase	AT1G63970	(Hsieh & Goodman, 2005)
**HDS**	hydroxy-2-methyl-2-(E)-butenyl 4-diphosphate synthase	AT5G60600	(Seemann *et al.*, 2005)
**HDR**	4-hydroxy-3-methylbut-2-en-1-yl diphosphate reductase	AT4G34350	(Guevara-García *et al.*, 2005)
**IPI1**	isopentenyl diphosphate:dimethylallyl diphosphate isomerase	AT3G02780	(Campbell *et al.*, 1998)
**IPI2**	isopentenyl diphosphate:dimethylallyl diphosphate isomerase	AT5G16440	(Campbell *et al.*, 1998)
**GPS**	geranyl diphosphate synthase	AT2G34630	(Bouvier *et al.*, 2000)
**FPS1**	farnesyl diphosphate synthase	AT4G17190	(Closa *et al.*, 2010)
**FPS2**	farnesyl diphosphate synthase	AT5G47770	(Closa *et al.*, 2010)
**GGPPS**	geranylgeranyl pyrophosphate synthase	AT1G49530	(Okada *et al.*, 2000)
**SQS**	squalene synthase 1	AT4G34640	(Nakashima *et al.*, 1995)
**SQE**	squalene epoxidase	AT1G58440	(Rasbery *et al.*, 2007)
**CAS**	cycloartenol synthase 1	AT2G07050	(Babiychuk *et al.*, 2008)
**LAS**	lanosterol synthase 1	AT3G45130	(Ohyama *et al.*, 2009)
**SMO1-1**	sterol 4-alpha-methyl oxidase 1-1	AT4G12110	(Darnet *et al.*, 2001)
**SMO2**	sterol 4-alpha-methyl oxidase 2	AT2G29390	(Darnet *et al.*, 2001)
**SMO1**	sterol 4-alpha-methyl oxidase 1	AT1G07420	(Darnet *et al.*, 2001)
**CPI1**	cyclopropyl isomerase	AT5G50375	(Lovato *et al.*, 2000)

**FK**	sterol C-14 reductase (Fackel)	AT3G52940	(Schrick *et al.*, 2004)
**CYP51**	cytochrome P450 51	AT1G11680	(Kim *et al.*, 2005; Kim *et al.*, 2010)
**HYD1**	C-8 sterol isomerase (Hydra)	AT1G20050	(Souter *et al.*, 2002)
**SMT1**	sterol methyltransferase 1	AT5G13710	(Carland *et al.*, 2010)
**SMT2**	sterol methyltransferase 2	AT1G20330	(Carland *et al.*, 2010)
**SMT3**	sterol methyltransferase 3	AT1G76090	(Carland *et al.*, 2010)
**DWF7**	delta7 sterol C-5 desaturation (Dwarf7)	AT3G02580	(Choe *et al.*, 1999b)
**DWF7-2**	delta7 sterol C-5 desaturation (Dwarf7-2)	AT3G02590	(Choe *et al.*, 1999b)
**DWF5**	sterol delta7 reductase (Dwarf5)	AT1G50430	(Lecain *et al.*, 1996)
**DWF1**	(24)-sterol reductase (Dwarf1)	AT3G19820	(Klahre *et al.*, 1998; Choe *et al.*, 1999a)
**DET2**	steroid-5-alpha-reductase (De-etiolated)	AT2G38050	(Chory *et al.*, 1991; Fujioka *et al.*, 1997)

Table S5. Primers used in these experiments.

Primer	Oligonucleotide Sequence 5'–3'	Product Size (bp)
**HMGS-g249-F**	CTTCAGATGCCTCATCGTGC	
**HMGS-g249-R**	TCTTCCCTAGATATGGGTCCGT	225
**GPS-g8423-F**	CAGGCAGGCGTGTTGAGC	
**GPS-g8423-R**	GGTTGCGGCGTGGTAGTC	143
**IPI-g7649-F**	AAGCCAATCAAAGCCATCTCC	
**IPI-g7649-R**	CATCGTCGCCAAGTTCTGC	208
**DXS-g6105-F**	GAGATTGCGACGGAAGATAGG	
**DXS-g6105-R**	CGCTGCATAAACGTGCTGTA	203
**DXR-g3685-F**	ATGAGCAGCCTCGTACAAAATAT	
**DXR-g3685-R**	CAGCAAGATGCGTGGGTCG	153
**CMS-g4346-F**	CCCAAACCGAATGAGGAAA	
**CMS-g4346-R**	CCAGCAAGAAGCACAACAGAG	252
**CMK-g9877-F**	CGCCTTTGGGGACACTTT	
**CMK-g9877-R**	GAGTTGGTTGGCTGCGAATA	92
**MDS-g3364-F**	TCTGCTGCCCACTCCGACG	
**MDS-g3364-R**	CACATTCCCAATTTTGTACCCTC	192
**HDS-g5186-F**	GGACGGATTAGGAGACGGG	
**HDS-g5186-R**	GCTGGGGCACGAGACAAA	133
**HDR-g6910-F**	CGTCGCATCATTCAATCGC	
**HDR-g6910-R**	CCCCAACAGAACCCGTAGC	185
**SQS-g8550-F**	AATGGCAACGGTTATAGGGAC	
**SQS-g8550-R**	AACTCGCAACGCACAGAAGA	123
**FK-g6702-F**	AGCCTAAGCAGATCAAGGTGAAGA	
**FK-g6702-R**	CGTAGTTTGGGCAGCAGTAGTAAA	174
**CYP51-g4528-F**	TGTCTTTACCGTCAACCTTTTCA	
**CYP51-g4528-R**	TCGGGTCACTGTCATACACTACTC	164
**SMT1-g7669-F**	AGCCAAGATCACAGGTGTAACCA	
**SMT1-g7669-R**	AACGAGCAGTCGGGAAACG	138
**SMT2-g7986-F**	CAGTCCTTTCACTTTGCTCCTCG	
**SMT2-g7986-R**	CATGCCTGGCTTCAACCCTAA	105
**DWF7-g9730-F**	CTTTTGACTTACCTTCGGGACTTG	
**DWF7-g9730-R**	GGGCTAACTGAATCTGATCCTTGA	166

**DWF5-g9012-F**	GAGCGTGACCCTGCAACTCG	
**DWF5-g9012-R**	CCAGGCAGCCCCAACAAA	119
**DWF1-g2385-F**	CAAGTTTGGCGGGTTTGTCA	
**DWF1-g2385-R**	CCCAGTCCTCTGCCTTTCGA	131
**PSY-g6239-F**	CGAGAAGAAGAAAGGGAGACGG	
**PSY-g6239-R**	CAGATGAAGCGACCAAGAGGAC	171
**ACS-g5570-F**	TCCCTACGCCCTGTCGCT	
**ACS-g5570-R**	CGCAAAATGTATTCCGTCTCG	137
**ACS-g1468-F**	GGGTGGAGGAGGTGATTGTG	
**ACS-g1468-R**	GGCGAAAGTGGCTTCAGTG	146
**ACC-g7511-F**	CGAGAACCTGCCGTTGATG	
**ACC-g7511-R**	GGCGGGATGTAGACGAAGAC	147
**ACC-g1005-F**	GGAAAGCAGGCAAGGTCG	
**ACC-g1005-R**	GATCCGTCGGGCAGAAGA	278
**ACLY-g8770-F**	GAACATCAAGGAGATTAGCCAGAG	
**ACLY-g8770-R**	GCGTGTAGCTCGTTCGTCAAG	93
**ME-g2252-F**	AGGGATGAACTACGAAATGGC	
**ME-g2252-R**	TGGTGGGGTTGGACAAGG	278
**ME-g3463-F**	TAAAATCGGCAGCAGCAATG	
**ME-g3463-R**	AGGGGTTCCTACAGATGTCCC	65
**FAS I-g927-F**	TGAAGCCCCATTGGTAGTCG	
**FAS I-g927-R**	GACCTAAGTCCCGCAAAATCTC	296
**FAE-g5258-F**	TGAGTTCCACCAAGTCTTCCAA	
**FAE-g5258-R**	ACCCGCATCGCTCCAATC	267
**FAE-g6504-F**	CTTCTCAGAGGCGGCACAA	
**FAE-g6504-R**	GAGCGTCAGATTCCAGTAGGC	253
**FAD-g1678-F**	CACAGACTTCATCTACAAGCATCC	
**FAD-g1678-R**	TCCACCTTATTTTCCCGCATA	282
**FAD-g7957-F**	GAGAAATGCTTCCAGAAATCCC	
**FAD-g7957-R**	GCGTGGCAAATGTGACCG	248

References

Armbrust EV, Berges JA, Bowler C, *et al*. 2004. The genome of the diatom *Thalassiosira pseudonana*: ecology, evolution, and metabolism. *Science* **306**(5693): 79-86.

Babiychuk E, Bouvier-Navé P, Compagnon V, Suzuki M, Muranaka T, Van Montagu M, Kushnir S, Schaller H. 2008. Allelic mutant series reveal distinct functions for Arabidopsis cycloartenol synthase 1 in cell viability and plastid biogenesis. *Proceedings of the National Academy of Sciences* **105**(8): 3163-3168.

Blanc G, Agarkova I, Grimwood J, *et al*. 2012. The genome of the polar eukaryotic microalga *Coccomyxa subellipsoidea* reveals traits of cold adaptation. *Genome Biology* **13**(5): R39.

Blanc G, Duncan G, Agarkova I, *et al*. 2010a. The *Chlorella variabilis* NC64A genome reveals adaptation to photosymbiosis, coevolution with viruses, and cryptic sex. *Plant Cell* **22**(9): 2943-2955.

Blanc G, Duncan G, Agarkova I, *et al*. 2010b. The *Chlorella variabilis* NC64A genome reveals adaptation to photosymbiosis, coevolution with viruses, and cryptic sex. *The Plant Cell* **22**(9): 2943-2955.

Bouvier F, Suire C, D'Harlingue A, Backhaus RA, Camara B. 2000. Molecular cloning of geranyl diphosphate synthase and compartmentation of monoterpene synthesis in plant cells. *The Plant Journal* **24**(2): 241-252.

Bowler C, Allen AE, Badger JH, *et al*. 2008. The *Phaeodactylum* genome reveals the evolutionary history of diatom genomes. *Nature* **456**(7219): 239-244.

Campbell M, Hahn F, Poulter CD, Leustek T. 1998. Analysis of the isopentenyl diphosphate isomerase gene family from *Arabidopsis thaliana*. *Plant Molecular Biology* **36**(2): 323-328.

Carland F, Fujioka S, Nelson T. 2010. The Sterol methyltransferases SMT1, SMT2, and SMT3 influence Arabidopsis development through nonbrassinosteroid products. *Plant physiology* **153**(2): 741-756.

Carretero-Paulet L, Cairó A, Talavera D, *et al*. 2013. Functional and evolutionary analysis of DXL1, a non-essential gene encoding a 1-deoxy-D-xylulose 5-phosphate synthase like protein in Arabidopsis thaliana. *Gene* **524**(1): 40-53.

Choe S, Dilkes BP, Gregory BD, Ross AS, *et al*. 1999a. The Arabidopsis dwarf1 mutant is defective in the conversion of 24-methylenecholesterol to campesterol in brassinosteroid biosynthesis. *Plant Physiology* **119**(3): 897-908.

Choe S, Noguchi T, Fujioka S, *et al*. 1999b. The Arabidopsis dwf7/ste1 mutant is defective in the Δ7 sterol C-5 desaturation step leading to brassinosteroid biosynthesis. *The Plant Cell* **11**(2): 207-221.

Chory J, Nagpal P, Peto CA. 1991. Phenotypic and genetic analysis of det2, a new mutant that affects light-regulated seedling development in Arabidopsis. *The Plant Cell* **3**(5): 445-459.

Closa M, Vranová E, Bortolotti C, Bigler L, Arró M, Ferrer A, Gruissem W. 2010. The *Arabidopsis thaliana* FPP synthase isozymes have overlapping and specific functions in isoprenoid biosynthesis, and complete loss of FPP synthase activity causes early developmental arrest. *The Plant Journal* **63**(3): 512-525.

Cock JM, Sterck L, Rouze P, *et al*. 2010. The *Ectocarpus* genome and the independent evolution of multicellularity in brown algae. *Nature* **465**(7298): 617-621.

Cordier H, Karst F, Bergès T. 1999. Heterologous expression in *Saccharomyces cerevisiae* of an *Arabidopsis thaliana* cDNA encoding mevalonate diphosphate decarboxylase. *Plant Molecular Biology* **39**(5): 953-967.

Darnet S, Bard M, Rahier A. 2001. Functional identification of sterol-4α-methyl oxidase cDNAs from *Arabidopsis thaliana* by complementation of a yeast *erg25* mutant lacking sterol-4α-methyl oxidation. *Febs Letters* **508**(1): 39-43.

Enjuto M, Balcells L, Campos N, Caelles C, Arró M, Boronat A. 1994. *Arabidopsis thaliana* contains two differentially expressed 3-hydroxy-3-methylglutaryl-CoA reductase genes, which encode microsomal forms of the enzyme. *Proceedings of the National Academy of Sciences* **91**(3): 927-931.

Fujioka S, Li J, Choi YH, Seto H, Takatsuto S, Noguchi T, Watanabe T, Kuriyama H, Yokota T, Chory J, Sakurai A. 1997. The Arabidopsis deetiolated2 mutant is blocked early in brassinosteroid biosynthesis. *The Plant Cell Online* **9**(11): 1951-1962.

Garcia-Hernandez M, Berardini T, Chen G, *et al*. 2002. TAIR: a resource for integrated Arabidopsis data. *Functional & Integrative Genomics* **2**(6): 239-253.

Guevara-García A, San Román C, Arroyo A, Cortés ME, de la Luz Gutiérrez-Nava M, León P. 2005. Characterization of the Arabidopsis clb6 mutant illustrates the importance of posttranscriptional regulation of the methyl-d-erythritol 4-phosphate pathway. *The Plant Cell* **17**(2): 628-643.

Hsieh M-H, Goodman HM. 2005. The Arabidopsis IspH homolog is involved in the plastid nonmevalonate pathway of isoprenoid biosynthesis. *Plant physiology* **138**(2): 641-653.

Initiative TAG. 2000. Analysis of the genome sequence of the flowering plant *Arabidopsis thaliana*. *Nature* **408**(6814): 796-815.

Jin H, Song Z, Nikolau BJ. 2012. Reverse genetic characterization of two paralogous acetoacetyl CoA thiolase genes in Arabidopsis reveals their importance in plant growth and development. *The Plant Journal* **70**(6): 1015-1032.

Kim HB, Lee H, Oh CJ, Lee H-Y, Eum HL, Kim HS, Hong YP, Lee Y, Choe S, An CS, Choi SB. 2010. Postembryonic seedling lethality in the sterol-deficient Arabidopsis cyp51A2 mutant is partially mediated by the composite action of ethylene and reactive oxygen species. *Plant Physiology* **152**(1): 192-205.

Kim HB, Schaller H, Goh C-H, Kwon M, Choe S, An CS, Durst F, Feldmann KA, Feyereisen R. 2005. Arabidopsis cyp51 mutant shows postembryonic seedling lethality associated with lack of membrane integrity. *Plant Physiology* **138**(4): 2033-2047.

Klahre U, Noguchi T, Fujioka S, Takatsuto S, Yokota T, Nomura T, Yoshida S, Chua N-H. 1998. The Arabidopsis DIMINUTO/DWARF1 gene encodes a protein involved in steroid synthesis. *The Plant Cell* **10**(10): 1677-1690.

Lecain E, Chenivesse X, Spagnoli R, Pompon D. 1996. Cloning by metabolic interference in yeast and enzymatic characterization of *Arabidopsis thaliana* sterol 7-reductase. *Journal Of Biological Chemistry* **271**(18): 10866-10873.

Lichtenthaler HK. 1999. The 1-deoxy-D-xylulose-5-phosphate pathway of isoprenoid biosynthesis in plants. *Annual review of plant biology* **50**(1): 47-65.

Lovato MA, Hart EA, Segura MJR, Giner J-L, Matsuda SPT. 2000. Functional cloning of an *Arabidopsis thaliana* cDNA encoding cycloeucalenol

cycloisomerase. *Journal Of Biological Chemistry* **275**(18): 13394-13397.

Lumbreras V, Campos N, Boronat A. 1995. The use of an alternative promoter in the *Arabidopsis thaliana* HMG1 gene generates an mRNA that encodes a novel 3-hydroxy-3-methylglutaryl coenzyme A reductase isoform with an extended N-terminal region. *The Plant Journal* **8**(4): 541-549.

Matsuzaki M, Misumi O, Shin-i T, *et al*. 2004. Genome sequence of the ultrasmall unicellular red alga *Cyanidioschyzon merolae* 10D. *Nature* **428**(6983): 653-657.

Merchant SS, Prochnik SE, Vallon O, *et al*. 2007. The *Chlamydomonas* genome reveals the evolution of key animal and plant functions. *Science* **318**(5848): 245-250.

Montamat F, Guilloton M, Karst F, Delrot S. 1995. Isolation and characterization of a cDNA encoding *Arabidopsis thaliana* 3-hydroxy-3-methylglutaryl-coenzyme A synthase. *Gene* **167**(1–2): 197-201.

Nakashima T, Inoue T, Oka A, Nishino T, Osumi T, Hata S. 1995. Cloning, expression, and characterization of cDNAs encoding *Arabidopsis thaliana* squalene synthase. *Proceedings of the National Academy of Sciences* **92**(6): 2328-2332.

Nieto B, Forés O, Arró M, Ferrer A. 2009. Arabidopsis 3-hydroxy-3-methylglutaryl-CoA reductase is regulated at the post-translational level in response to alterations of the sphingolipid and the sterol biosynthetic pathways. *Phytochemistry* **70**(1): 53-59.

Nozaki H, Takano H, Misumi O, *et al*. 2007. A 100%-complete sequence reveals unusually simple genomic features in the hot-spring red alga *Cyanidioschyzon merolae*. *BMC Biology* **5**(1): 1-8.

Ohyama K, Suzuki M, Kikuchi J, Saito K, Muranaka T. 2009. Dual biosynthetic pathways to phytosterol via cycloartenol and lanosterol in Arabidopsis. *Proceedings of the National Academy of Sciences* **106**(3): 725-730.

Okada K, Saito T, Nakagawa T, Kawamukai M, Kamiya Y. 2000. Five geranylgeranyl diphosphate synthases expressed in different organs are localized into three subcellular compartments in Arabidopsis. *Plant physiology* **122**(4): 1045-1056.

Palenik B, Grimwood J, Aerts A, *et al*. 2007. The tiny eukaryote *Ostreococcus* provides genomic insights into the paradox of plankton speciation. *Proceedings of the National Academy of Sciences* **104**(18): 7705-7710.

Phillips MA, León P, Boronat A, Rodríguez-Concepción M. 2008. The plastidial MEP pathway: unified nomenclature and resources. *Trends in plant science* **13**(12): 619-623.

Prochnik SE, Umen J, Nedelcu AM, *et al*. 2010. Genomic analysis of organismal complexity in the multicellular green alga *Volvox carteri*. *Science* **329**(5988): 223-226.

Rasbery JM, Shan H, LeClair RJ, Norman M, Matsuda SPT, Bartel B. 2007. *Arabidopsis thaliana* squalene epoxidase 1 is essential for root and seed development. *Journal Of Biological Chemistry* **282**(23): 17002-17013.

Riou C, Tourte Y, Lacroute F, Karst F. 1994. Isolation and characterization of a cDNA encoding *Arabidopsis thaliana* mevalonate kinase by genetic complementation in yeast. *Gene* **148**(2): 293-297.

Rohdich F, Wungsintaweekul J, Eisenreich W, Richter G, Schuhr CA, Hecht S, Zenk MH, Bacher A. 2000. Biosynthesis of terpenoids: 4-Diphosphocytidyl-2C-methyl-d-erythritol synthase of *Arabidopsis thaliana*.

Proceedings of the National Academy of Sciences **97**(12): 6451-6456.

Schrick K, Fujioka S, Takatsuto S, Stierhof Y-D, Stransky H, Yoshida S, Jürgens G. 2004. A link between sterol biosynthesis, the cell wall, and cellulose in Arabidopsis. *The Plant Journal* **38**(2): 227-243.

Schwender J, Müller C, Zeidler J, Lichtenthaler HK. 1999. Cloning and heterologous expression of a cDNA encoding 1-deoxy-D-xylulose-5-phosphate reductoisomerase of *Arabidopsis thaliana*. *FEBS letters* **455**(1): 140-144.

Seemann M, Wegner P, Schünemann V, Bui B, Wolff M, Marquet A, Trautwein A, Rohmer M. 2005. Isoprenoid biosynthesis in chloroplasts via the methylerythritol phosphate pathway: the (E)-4-hydroxy-3-methylbut-2-enyl diphosphate synthase (GcpE) from *Arabidopsis thaliana* is a [4Fe–4S] protein. *JBIC Journal of Biological Inorganic Chemistry* **10**(2): 131-137.

Souter M, Topping J, Pullen M, Friml J, Palme K, Hackett R, Grierson D, Lindsey K. 2002. Hydra mutants of Arabidopsis are defective in sterol profiles and auxin and ethylene signaling. *The Plant Cell Online* **14**(5): 1017-1031.

Wang D, Ning K, Li J, Hu Q, Xu J. 2013. Genomic foundation of oil production in oleaginous microalgae. *Plos Genetics* ***in press***.

Worden AZ, Lee JH, Mock T, *et al*. 2009. Green evolution and dynamic adaptations revealed by genomes of the marine picoeukaryotes Micromonas. *Science* **324**(5924): 268-272.
